# Supplementary material for: Validation of maternal recall of number of antenatal care visits attended in rural Southern Nepal: a longitudinal cohort study
Source: BMJ Open. 2023 Dec 9;13(12):e079029. doi: 10.1136/bmjopen-2023-079029 (PMC10729047; doi:10.1136/bmjopen-2023-079029)

SUPPLEMENTAL TABLES AND FIGURES

Supplemental Table 1. Characteristics of participants with postpartum interview

Characteristic	Observed all ANC visits (N=204)		Received ANC between observations (N=230)		Two sample t-test p-value	Total (N=434)	
	Mean (SD)	Range	Mean (SD)	Range		Mean (SD)	Range
Woman's age, years	22.8 (4.3)	16 to 41	22.3 (4.0)	16 to 35	0.184	22.5 (4.2)	16 to 41
Total number of ANC visits observed	3.2 (2.0)	1 to 10	5.6 (2.3)	2 to 14	<0.01	4.5 (2.5)	1 to 14
Number of months between last ANC observation and postpartum interview	11.6 (3.3)	3 to 22	9.1 (2.5)	3 to 17	<0.01	10.3 (3.2)	3 to 22

	Observed all ANC visits (N=204)		Received ANC between observations (N=230)		Chi-square p-value	Total (N=434)	
Most recent pregnancy outcome							
Miscarriage/abortion	27	13.2%	1	0.4%	<0.01	28	6.5%
Stillbirth	3	1.5%	1	0.4%		4	0.9%
At least one live birth	174	85.3%	228	99.1%		402	92.6%
4 quantiles of SES							
1	94	46.1%	73	31.7%	<0.01	167	38.5%
2	39	19.1%	35	15.2%		74	17.1%
3	48	23.5%	84	36.5%		132	30.4%
4	23	11.3%	38	16.5%		61	14.1%
Is this the woman's first pregnancy?							
No	153	75.0%	145	63.0%	<0.01	298	68.7%
Yes	51	25.0%	85	37.0%		136	31.3%
Did the woman receive any years of education?							
No	139	68.1%	120	52.2%	<0.01	259	59.7%
Yes	65	31.9%	110	47.8%		175	40.3%
Trimester at enrollment							
1-3 months	81	39.7%	109	47.4%	0.1	190	43.8%
4-6 months	117	57.4%	119	51.7%		236	54.4%
7-9 months	6	2.9%	2	0.9%		8	1.8%

Supplemental Figure 1. Agreement between numbers of ANC visits observed at health posts and maternal report at postpartum interview in (A) the entire cohort (n = 402) and (B) the subgroup with all ANC observed (n = 174).

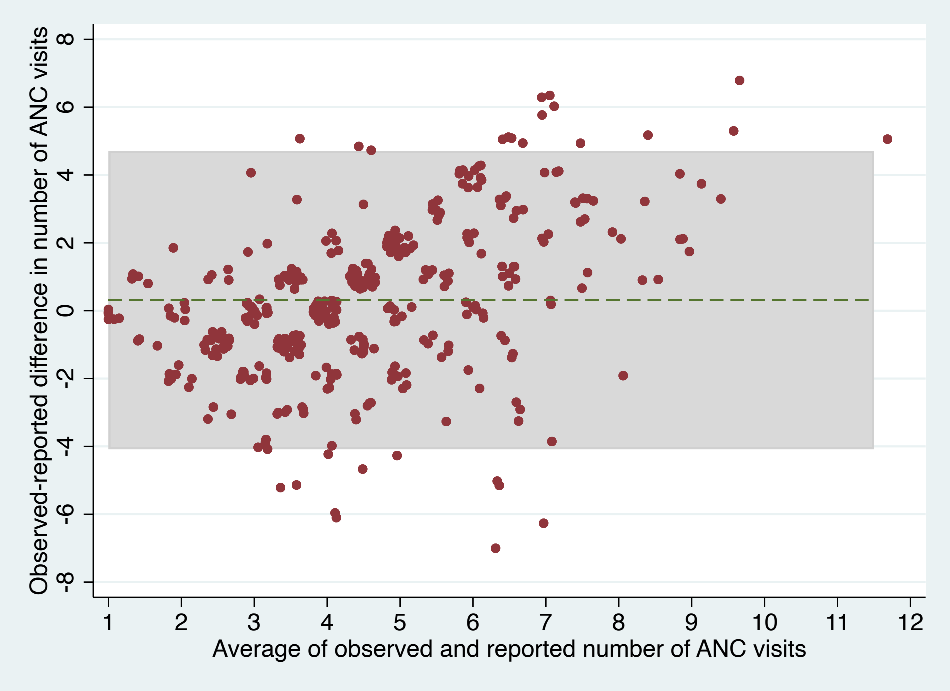

(A)

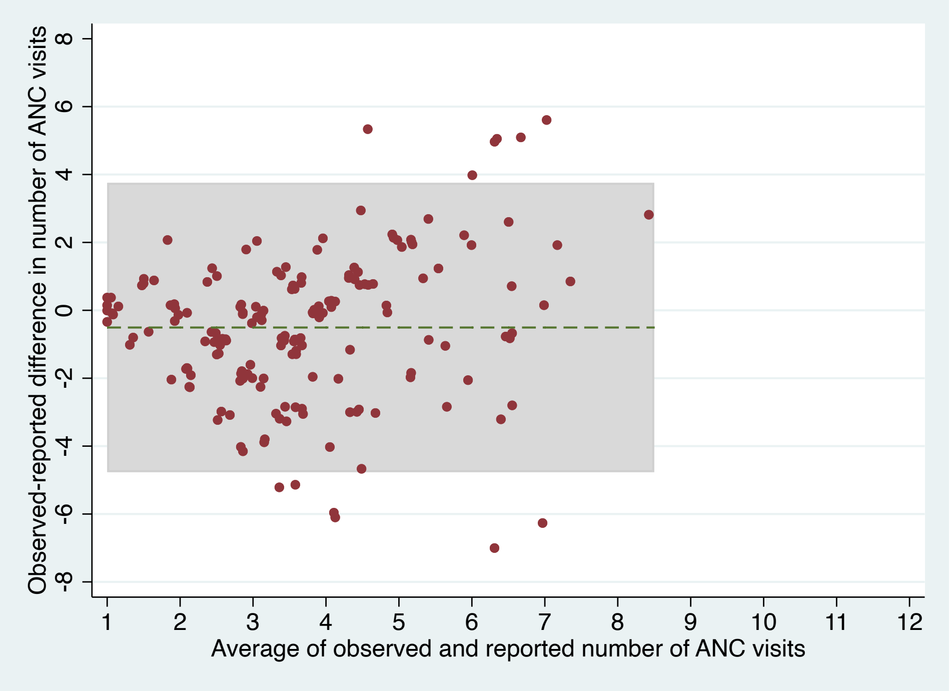

(B)

Supplemental Figure 2. Histogram of observed number of ANC visits (n = 402).

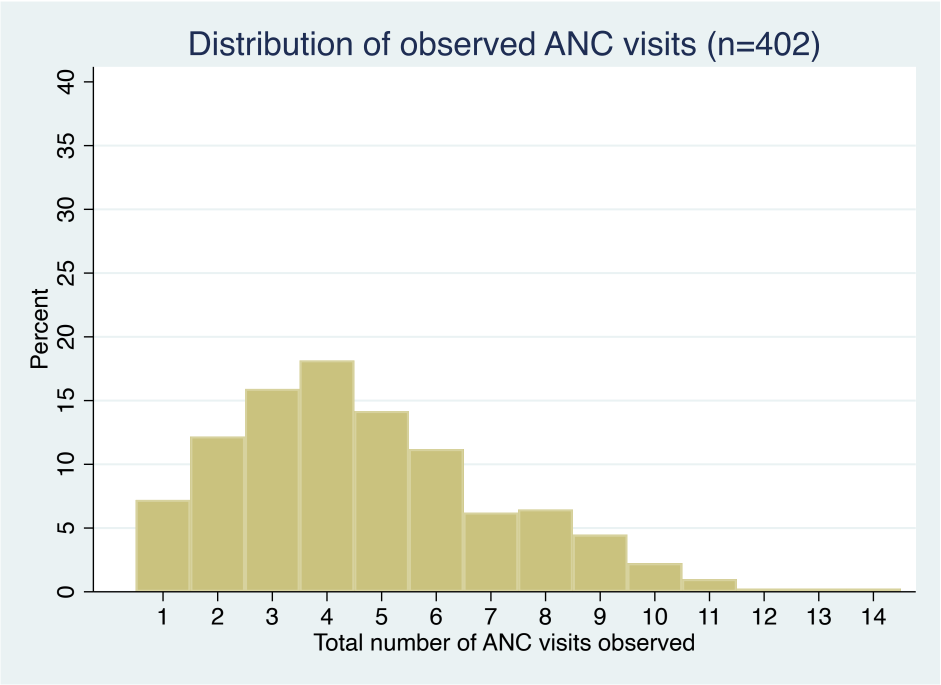

Supplemental Figure 3. Histogram of reported number of ANC visits (n = 402).

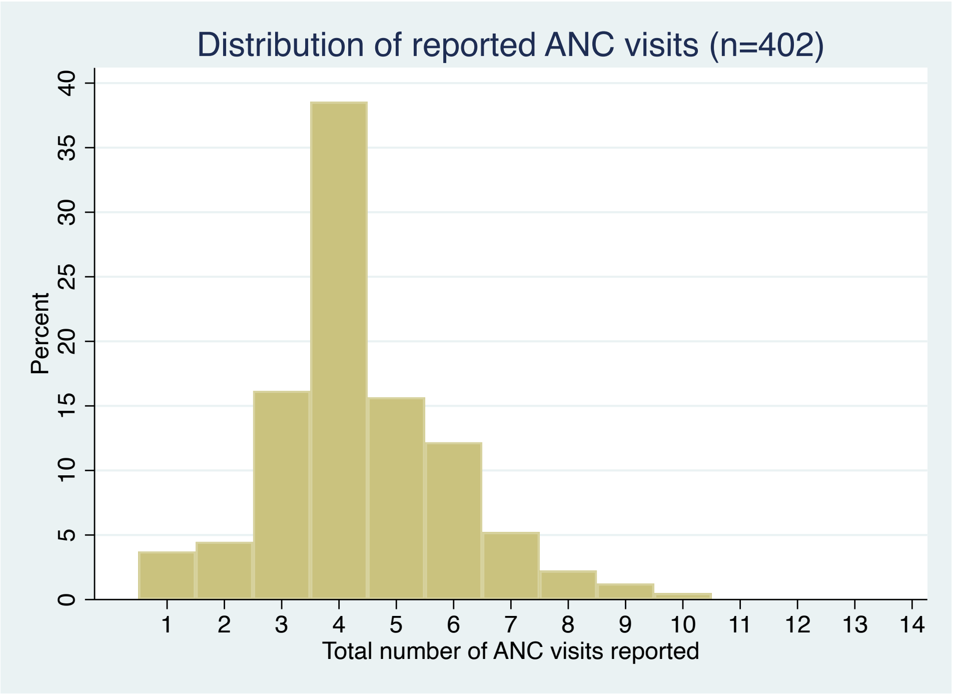

Supplement: Supplementary data [file bmjopen-2023-079029supp001.pdf]
