# Supplementary material for: Validation of maternal recall of number of antenatal care visits attended in rural Southern Nepal: a longitudinal cohort study
Source: BMJ Open. 2023 Dec 9;13(12):e079029. doi: 10.1136/bmjopen-2023-079029 (PMC10729047; doi:10.1136/bmjopen-2023-079029)
Supplement: Author's manuscript [file bmjopen-2023-079029.draft_revisions.pdf]

BMJ Open

BMJ Open is committed to open peer review. As part of this commitment we make the peer review history of every article we publish publicly available.

When an article is published we post the peer reviewers' comments and the authors' responses online. We also post the versions of the paper that were used during peer review. These are the versions that the peer review comments apply to.

The versions of the paper that follow are the versions that were submitted during the peer review process. They are not the versions of record or the final published versions. They should not be cited or distributed as the published version of this manuscript.

BMJ Open is an open access journal and the full, final, typeset and author-corrected version of record of the manuscript is available on our site with no access controls, subscription charges or pay-per-view fees (http://bmjopen.bmj.com).

If you have any questions on BMJ Open's open peer review process please email info.bmjopen@bmj.com

BMJ Open

Validation of maternal recall of antenatal care visits in rural Nepal

Journal:	*BMJ Open*
Manuscript ID	bmjopen-2023-079029
Article Type:	Original research
Date Submitted by the Author:	19-Aug-2023

Complete List of Authors:	Xie, Xinyu; Johns Hopkins University Bloomberg School of Public Health, International Health<br>Munos, Melinda; Johns Hopkins University, Bloomberg School of Public Health, Department of International Health<br>Plama, Tsering; Johns Hopkins University Bloomberg School of Public Health, Department of International Health<br>Bryce, Emily; Johns Hopkins University Bloomberg School of Public Health,<br>Khatry, Subarna; Nepal Nutrition Intervention Project - Sarlahi<br>LeClerq, Steven C.; Johns Hopkins Bloomberg School of Public Health, Department of International Health; Nepal Nutrition Intervention Project<br>Katz, Joanne; Johns Hopkins University,

Keywords:	Quality in health care < HEALTH SERVICES ADMINISTRATION & MANAGEMENT, Patient Reported Outcome Measures, Surveys and Questionnaires

SCHOLARONE™
Manuscripts

I, the Submitting Author has the right to grant and does grant on behalf of all authors of the Work (as defined in the below author licence), an exclusive licence and/or a non-exclusive licence for contributions from authors who are: i) UK Crown employees; ii) where BMJ has agreed a CC-BY licence shall apply, and/or iii) in accordance with the terms applicable for US Federal Government officers or employees acting as part of their official duties; on a worldwide, perpetual, irrevocable, royalty-free basis to BMJ Publishing Group Ltd ("BMJ") its licensees and where the relevant Journal is co-owned by BMJ to the co-owners of the Journal, to publish the Work in this journal and any other BMJ products and to exploit all rights, as set out in our licence.

The Submitting Author accepts and understands that any supply made under these terms is made by BMJ to the Submitting Author unless you are acting as an employee on behalf of your employer or a postgraduate student of an affiliated institution which is paying any applicable article publishing charge ("APC") for Open Access articles. Where the Submitting Author wishes to make the Work available on an Open Access basis (and intends to pay the relevant APC), the terms of reuse of such Open Access shall be governed by a Creative Commons licence – details of these licences and which Creative Commons licence will apply to this Work are set out in our licence referred to above.

Other than as permitted in any relevant BMJ Author's Self Archiving Policies, I confirm this Work has not been accepted for publication elsewhere, is not being considered for publication elsewhere and does not duplicate material already published. I confirm all authors consent to publication of this Work and authorise the granting of this licence.

For peer review only

Title:

Validation of maternal recall of antenatal care visits in rural Nepal

Authors and Institutions: Xinyu Xie, Melinda Munos, Tsering Pema Lama, Emily Bryce, Subarna Khatry, Steven LeClerq, Joanne Katz

Keywords: Nepal, antenatal care, recall, validity, coverage, measurement

Correspondence to: Xinyu Xie; 615 N. Wolfe Street, Baltimore, MD 21205; evaxie.shchn@gmail.com, +1 443-839-5016

For peer review only

ABSTRACT

Objectives: This study aimed to examine the validity of maternal recall of total number of antenatal care (ANC) visits during pregnancy and factors associated with the accuracy of maternal recall.

Design: This study was a longitudinal cohort study conducted from December 2018 through November 2020.

Setting: Five government health posts in the Sarlahi District of Southern Nepal

Participants: 402 pregnant women between ages of 15 and 49 who presented for their first ANC visit at the study health posts

Main Outcomes: The observed number of total ANC visits (gold standard) and the reported number of total ANC visits at the postpartum interview (maternal recall)

Results: On average, women in the study who had a live birth attended 4.7 ANC visits. About 65% of them attended four or more ANC visits during pregnancy as recommended by the Nepal government, and 38.3% of maternal report matched the categorical ANC visits as observed by the gold standard. The individual validity was poor to moderate, with the highest AUC being 0.69 (95% CI: 0.65, 0.74) in the 1-3 visits group. Population bias was observed in the 1-3 visits and 4 visits groups, where 1-3 visits were underreported (IF: 0.69) and 4 ANC visits were highly overreported (IF: 2.12). The binary indicator ANC4+ showed better population-level validity (AUC: 0.69; IF: 1.17) compared with multi-categorized ANC visits. Report accuracy was not associated with maternal characteristics but was related to ANC frequency. Women who attended more ANC visits were less likely to correctly report their total number of visits.

Conclusion: Maternal report of total number of ANC visits during pregnancy may not be a valid indicator for measuring ANC coverage. Improvements are needed to measure the frequency of ANC visits so that it can produce high-quality data for tracking population health, service coverage, and national program planning.

For peer review only

ARTICLE SUMMARY

Strengths and limitations of this study

- The gold standard was established using direct observation by trained field workers, thus eliminating the risk of recall bias.
- The study observers were all trained to reach a standard level of validity before working at the study sites, which provides a more objective and reliable source for verification than secondary databases.
- The study had an appropriate length of recall period comparing to other validation studies who use recall periods of less than six months or even exit interviews to validate maternal report.
- The study only considered women who presented for their first ANC at public health posts for the feasibility of data collection.
- Women who visited facilities other than the study health posts were not observed but were asked to recall how many other ANC visits they attended.

For peer review only

INTRODUCTION

The United Nations Inter-Agency Group estimated that in 2020 the global maternal mortality ratio (MMR) was 223 deaths per 100,000 live births, and UNICEF reported an average global neonatal mortality rate (NMR) of 18 deaths per 1,000 live births in 2021 [1,2]. Maternal and neonatal mortality remains an issue that differentially impacts developed and developing countries. According to the World Health Organization (WHO), 94% of all maternal deaths in 2017 occurred in low- and middle-income countries (LMICs), with 86% taking place in sub-Saharan Africa and South Asia [3]. Sub-Saharan Africa and South Asia also have the highest NMR among all regions (27 and 23 deaths per 1,000 live births respectively in 2021) [2].

Antenatal care (ANC) plays an important role in maternal and neonatal health. By providing health contacts with the mother at key points in the continuum of care, quality ANC greatly reduces the risk of maternal mortality through preventive and promotive care and early detection and treatment of pregnancy-related complications, improving the survival and health of newborns [3-5]. In 2002, the WHO introduced the focused ANC (FANC) model consisting of at least four ANC visits during pregnancy, but the model had limited effectiveness on service uptake and was associated with increased underutilization in resource-limited settings [6-8]. The WHO published an updated guideline on ANC in 2016 changing the number of ANC visits from four times to a minimum of eight contacts [6].

The Ministry of Health and Population (MoHP) in Nepal followed the FANC model with at least four ANC visits at the 4th, 6th, 8th, and 9th month of gestation when they conducted the Nepal Demographic and Health Survey (DHS) in 2016 [9]. To improve the utilization of ANC, the Nepal government started a national Safe Delivery Incentive Program, or Aama Program in Nepali [10]. This program provides monetary incentives to women who completed at least four ANC visits as suggested by the MoHP and women who delivered at health facilities by skilled birth attendants [10]. However, studies have found that recipients of the incentives were disproportionally wealthy families that had

For peer review only

more access to health services and policy information, and the program had limited effect on ANC utilization in rural areas [11,12]. The MoHP published the National Medical Standard for Maternal and Newborn Care in 2020, stating that Nepal now recommends the new WHO eight contacts of ANC approach [13].

According to the 2022 Nepal DHS, the ANC service utilization rate was 94% for at least one ANC visit among 15- to 49-year-old women who had a live or stillbirth within two years before the survey; 80% of women had four or more ANC visits during their latest pregnancy and 82% of women in rural regions had at least four ANC visits [14].

Nepal has a national household survey every five years to evaluate the national ANC coverage, and the frequency of ANC visits serves as an important indicator. However, the survey often takes place many years after a woman's pregnancy. It is unknown whether the woman can correctly recall the total number of ANC visits and provide accurate answers to the DHS question. Therefore, the validity of this question in such household surveys is unknown. Previous studies have investigated the validity of ANC coverage indicators like quality of care, nutritional interventions, nutrition counselling, and iron-folic acid supplementation in the same Nepal cohort, but the validity of frequency of ANC visits has not been explored [15-17]. The objective of this study is to examine the validity of maternal report of total number of ANC visits and factors associated with the accuracy of maternal report.

METHODS

Study site

This longitudinal cohort study was conducted from December 2018 to November 2020 within the study area of the Nepal Nutrition Intervention Project Sarlahi (NNIPS) located in the rural Sarlahi District of Southern Nepal. Sarlahi is a part of the Madhesh Province bordered to the west by the Bagmati River and to the south by the state of Bihar, India. Two municipalities (Haripur and Kabilasi) were chosen based on the census data and experiences from the local study team. Sarlahi district has a

For peer review only - http://bmjopen.bmj.com/site/about/guidelines.xhtml

female population of 379,973 and 47.8% of these are between 15 to 49 years of age [18]. Previous studies in the NNIPS area showed that women in Sarlahi district had an estimated pregnancy-related mortality ratio of 529 deaths per 100,000 live births in the period 2001-2006, which was almost twice of the national average [19]. Nepal DHS does not report maternal mortality ratios at the district level so there is no more recent comparable data. Approximately 60% of women in this area attended four or more ANC visits in the period 2010-2016, which is lower than the average among rural regions [15]. Five public health posts at Pharadwa, Laxmipur, Pidari, Piparya, and Kabilasi village development committees (VDCs)were designated to be the study sites because of their high attendance at ANC and accessibility to both the clients and the study team. VDCs have now been dissolved, but at that time VDCs were the smallest administrative unit in district where each VDC had nine wards.

Study population, design, and data collection

All pregnant women aged 15 years and older who lived in the NNIPS area who came for their first ANC visit to one of the five study health posts were eligible for the study. Women in the study were assumed to be married since it would be culturally inappropriate to ask about their marital status if were pregnant and seeking ANC. Women who were younger than 15 years old were not enrolled. Women were considered ineligible to participate if they had already attended ANC or an ultrasound appointment before recruitment because not all ANC visits would be observed by the study team. Those who planned on visiting other health facilities than the five study ones for ANC during pregnancy were also considered ineligible for the same reason. Women who planned on leaving the NNIPS area during the study period, or up until six months after delivery were excluded to prevent and minimize any loss to follow-up. Participants were consented at the enrollment visit and during the postpartum interview respectively.

The overall study approach is to assess the validity of maternal report by comparing the observed number of ANC visits (gold standard) to the answers provided by women in the six-month

For peer review only - http://bmjopen.bmj.com/site/about/guidelines.xhtml

postpartum interview. During the enrollment period, trained field workers collected the demographic data of eligible participants, such as women's age, gestational age, parity and education level. Once enrolled, the participants were asked to complete a follow-up survey at each of their ANC visits. Trained field workers recorded their presence at the ANC visit and asked them questions about any health-seeking behavior since the last visit. The follow-up form asked questions like "what is the location of your most recent ANC visit" to help determine if the woman attended any ANC that was not observed by the study team. These direct observations served as the "gold standard" for the validation analysis. A postpartum interview was conducted approximately six months after the woman's delivery to collect information on the ANC services they received during pregnancy. Some of the interview questions were constructed using the same language as the 2016 Nepal DHS. Specifically, the question about the number of ANC visits attended in the most recent pregnancy was identical to the question in the Nepal DHS ("How many times did you receive antenatal care during this pregnancy?"). The exact Nepali used in the Nepal DHS was used for this question. The interview also collected information on their socioeconomic status (SES) through questions about housing, household asset ownership, cooking fuels, and ownership of land and household goods.

Analysis

The study aimed to enroll 450 women to reach a sample size of 300, to estimate validation measures with sufficient precision (with prevalence of 50%, a 95% confidence interval would be 13% wide or +/- 6.5% points), accounting for women who did not have a live birth, those who may have gone elsewhere for some ANC visits and did not have all visits observed, and loss to follow-up. Eventually 441 women were enrolled in the study and 434 of them participated in the postpartum interview.

The gold standard of observed number of visits was compared to the maternal report of the number of ANC visits for the validity analysis. Since it was impractical to follow women everywhere throughout their pregnancy, the follow-up survey at each ANC visit collected information to determine

For peer review only

whether women received ANC at facilities other than the five designated health posts where observers were stationed. Participants were categorized into those who sought ANC elsewhere and those for whom all ANC was observed by the study team. In this way, a stricter gold standard was available for subgroup analysis.

The study cohort was categorized by the total number of ANC visits: 1-3 versus 4 or more (4+) visits; 1-3 visits, 4 visits, 5-6 visits, and more than 6 visits. Since the Nepal MoHP recommended four or more ANC visits during pregnancy at the time of the study, the 4+ visits group was designed to see the compliance of FANC model and test the validity of a binary ANC frequency indicator. Individual validity was evaluated through sensitivity, specificity, and area under the receiver operating characteristic curve (AUC). To calculate sensitivity and specificity, 2x2 tables were constructed. Each participant was assigned to a cell in the table based on whether their ANC visit number fell in the group according to the gold standard and the maternal report. The calculation of sensitivity and specificity is similar to that of a diagnostic test. AUC in this scenario represents the probability that a woman's report of number of ANC visits is consistent with the gold standard category. AUC is calculated as the area under the plot of sensitivity vs. (1-specificity) [20]. An AUC higher than 0.7 is considered as high individual-level accuracy; an AUC of 0.5 indicates that maternal report on the indicator is no better than a random guess [20]. Population-level validity was measured through the inflation factor (IF), which gives an estimate of the accuracy of the postpartum survey in reflecting the true coverage in the population. It is calculated as the study coverage measured from maternal report divided by the true population coverage value based on the gold standard. The study coverage can be calculated using the formula: $Pr=P(SN+SP-1)+(1-SP)$, where Pr is the study coverage, P is the true population coverage, SN is sensitivity, and SP is specificity [20]. An IF of 1.00 indicates perfect accuracy and an IF between 0.75 and 1.25 means there is low population bias [20].

For peer review only - http://bmjopen.bmj.com/site/about/guidelines.xhtml

Bivariate and multivariate log-binomial regression models were used to assess factors associated with accuracy of maternal report. The primary outcome, accuracy, is a dichotomous variable. Maternal report of the number of ANC visits either matched with the categorical number of ANC visits observed (the gold standard), indicating accuracy, or it did not match (not accurate). Relative risk of accurately reporting was calculated because accurate reports were not rare outcomes; 38% of women recalled the number of ANC visits accurately according to the categorical definition described previously (1-3 versus 4+ visits; or 1-3 visits, 4 visits, 5-6 visits, and more than 6 visits). Covariates related to maternal characteristics included maternal age, maternal education, number of prior live births, and household SES. All covariates were included in the adjusted model. Maternal age was dichotomized into younger or older than 25 years. Any education was compared to no education and any previous live birth was compared to no previous live birth. The household SES variable was constructed based on family-owned land, animals and household items and housing infrastructures like types of cooking fuels, toilet, and water sources. Housing characteristics were assigned scores and summed up for each woman. The total score was divided by the number of non-missing variables and separated into quartiles. Time between the postpartum interview and the last ANC observation was dichotomized to more or less than 1 year after examining its locally weighted scatterplot smoother (LOWESS) versus report accuracy. The observed total number of ANC visits was classified as 1-3, 4-7, and 8 or more using the LOWESS curve. Both LOWESS curves appeared linear in segments with a knot at approximately 1 year and knots at the 4th and 8th visits. A p-value less than 0.05 was considered statistically significant.

All analyses were conducted using Stata version 17.0 (StatCorp).

Patient and public involvement

Study participants were not involved in the design, recruitment, conduct, or dissemination of this research. The 28-item checklist used for direct observation of the first and all subsequent ANC visits was reviewed by a local community advisory board in Nepal before the start of the study, but the public

For peer review only - http://bmjopen.bmj.com/site/about/guidelines.xhtml

had no other part in the development or implementation of this study. There are no plans to disseminate results to the participants or community, aside from the local study staff who reside in the community.

RESULTS

Among the 441 women enrolled in the study, seven were lost to follow-up due to migration out of the study area and were not available for the postpartum interview. There was no difference between the background characteristics of the participants who were lost to follow-up and those who stayed in the study. Thirty-two women were excluded from the validation analysis because of their birth outcomes (not a live birth). At the time of the study, in the DHS, women with a pregnancy not resulting in a live birth were not asked the question about number of ANC visits (although more recent DHS do). 402 women met the Nepal DHS sampling criteria and were included in the analysis. Among the 402 women, 228 reported receiving ANC from non-study facilities, leaving 174 women with complete ANC observation by the study team. Figure 1 shows the flowchart of participants.

Table 1. Characteristics of participants with live births[1]

	Observed all ANC visits (N=174)		Received ANC between observations (N=228)		Two sample t-test p-value	Total (N=402)	
Characteristic	Mean (SD)	Range	Mean (SD)	Range		Mean (SD)	Range
Woman's age, years	22.7 (4.4)	16-41	22.3 (4.1)	16-35	0.318	22.5 (4.2)	16-41
Total number of ANC visits observed	3.4 (2.1)	1-10	5.6 (2.3)	2-14	<0.01	4.7 (2.5)	1-14
Number of months between last ANC observation and postpartum interview	11.2 (3.2)	3-21	9.1 (2.5)	3-17	<0.01	10.0 (3.0)	3-21

	Observed all ANC visits (N=174) n (%)	Received ANC between observations (N=228) n (%)	Chi-square p-value	Total (N=402) n (%)
4 quantiles of SES				
1	76 (43.7)	72 (31.6)	<0.01	148 (36.8)

For peer review only

For peer review only - http://bmjopen.bmj.com/site/about/guidelines.xhtml

2	36 (20.7)	35 (15.4)		71 (17.7)
3	43 (24.7)	83 (36.4)		126 (31.3)
4	19 (10.9)	38 (16.7)		57 (14.2)
Is this the woman's first pregnancy?				
No	133 (76.4)	143 (62.7)	<0.01	276 (68.7)
Yes	41 (23.6)	85 (37.3)		126 (31.3)
Did the woman receive any years of education?				
No	121 (69.5)	119 (52.2)	<0.01	240 (59.7)
Yes	53 (30.5)	109 (47.8)		162 (40.3)
Trimester at enrollment				
1-3 months	63 (36.2)	107 (46.9)	0.043	170 (42.3)
4-6 months	106 (60.9)	119 (52.2)		225 (56.0)
7-9 months	5 (2.9)	2 (0.9)		7 (1.7)
Frequency of ANC visits				
1-3 visits	103 (59.2)	39 (17.1)	<0.01	142 (35.3)
4 visits	25 (14.4)	48 (21.1)		73 (18.2)
5-6 visits	31 (17.8)	71 (31.1)		102 (25.4)
More than 6 visits	15 (8.6)	70 (30.7)		85 (21.1)

[1] ANC, antenatal care; SES, socioeconomic status.

Table 1 summarizes the maternal characteristics of women who attended the postpartum interview and had a live birth outcome. The age of women ranged from 16 to 41 years, with a mean age of 22.5. There was no significant age difference between women who sought ANC elsewhere and those who did not. The observed total number of ANC visits ranged from 1 to 14. On average, women attended 4.7 ANC visits during their pregnancy. The number of ANC visit was higher among women who sought ANC in non-study facilities. About 65% of women attended four or more ANC visits, the majority of which (72.7%) were women who reported receiving ANC from non-study clinics between observations at the study clinics. About 60% of women had not received any education. Women who received ANC from non-study clinics were more educated and had higher SES.

Bland-Altman plots were constructed to compare ANC visit frequencies as observed by the gold standard and reported by maternal recall (Supplemental Figure 1). In the entire cohort, the mean number of ANC visits observed was 4.7 (SD = 2.5), compared with the reported number of 4.4 visits (SD

For peer review only

= 1.6). We observed both over-reporting and under-reporting of number of ANC visits, relative to the number observed (Supplemental Figure 1A). Over-reporting was common among women who had fewer ANC visits, while under-reporting was common among higher ANC frequencies. In the subgroup of women whose ANC was fully observed (Supplemental Figure 1B), the observed mean of total visits was 3.4 (SD = 2.1), while the reported mean was 4.0 (SD = 1.7). The distribution of observed total number of visits was positively skewed, with a long tail of women receiving 8+ visits, while the reported visits were more normally distributed (Supplemental Figure 2 and 3). The disparity between the observed and reported distributions implied that women who had less or more than four ANC visits tended to report that they had four visits during pregnancy.

A total of 402 women with live births were included in the validity analysis, as per the Nepal DHS protocol. The validation results from the 402 women are shown in Table 2A. The binary indicator of 4+ visits, which is used for global reporting and tracking, had a sensitivity of 89.2% (95% CI: 84.8, 92.7%) and a specificity of 49.3% (95% CI: 40.8, 57.8%). It showed a moderate level of individual validity (AUC: 0.69; 95% CI:0.65, 0.74) and low population bias (IF:1.17). The categorized visit groups, on the other hand, demonstrated poorer validity than the binary indicator in terms of sensitivity, AUC, and IF. In general, sensitivity was low and had a declining trend with more ANC visits. The 1-3 visits category had the highest sensitivity score of 49.3% (95% CI: 40.8, 57.8%). Specificity ranged from 63.5% (95% CI: 58.1, 68.7%) in the 4 visits group to 94.0% (95%CI: 90.8, 96.4%) in the more than 6 visits group. Only the 1-3 visits group showed a moderate level of individual validity (AUC: 0.69; 95% CI: 0.65, 0.74), while other groups all had AUC less than 0.6 but barely better than a random guess. Population bias was common in all groups except the 5-6 visits group (IF: 1.10). There was high overestimation of ANC visit frequency in the 4 visits group (IF: 2.12) and underestimation in the other two groups. However, specificity of ANC categories was much better than that of the binary indicator. Specificity ranged from 63.5% (95% CI: 58.1, 68.7%) in the 4 visits group to 94.0% (95%CI: 90.8, 96.4%) in the more than 6 visits group.

For peer review only - http://bmjopen.bmj.com/site/about/guidelines.xhtml

For peer review only

Table 2. Validation of maternal report of ANC visits[1]

A. Among women with livebirths (N = 402)

Gold standard vs. reported	Sensitivity (95% CI), %	Specificity (95% CI), %	AUC (95% CI)	"True" coverage (95% CI), %	Estimated survey coverage, %	Inflation factor
No. of ANC visits						
FANC model (4 or more)	89.2 (84.8, 92.7)	49.3 (40.8, 57.8)	0.69 (0.65, 0.74)	64.7 (59.8, 69.4)	75.6	1.17
1 to 3	49.3 (40.8, 57.8)	89.2 (84.8, 92.7)	0.69 (0.65, 0.74)	35.3 (30.6, 40.2)	24.4	0.69
4	47.9 (36.1, 60.0)	63.5 (58.1, 68.7)	0.56 (0.49, 0.62)	18.2 (14.5, 22.3)	38.6	2.12
5 to 6	30.4 (21.7, 40.3)	73.0 (67.6, 77.9)	0.52 (0.47, 0.57)	25.4 (21.2, 29.9)	27.9	1.10
More than 6	21.2 (13.1, 31.4)	94.0 (90.8, 96.4)	0.58 (0.53, 0.62)	21.1 (17.3, 25.5)	9.2	0.44

B. Among women with livebirths and fully observed (N = 174)

Gold standard vs. reported	Sensitivity (95% CI), %	Specificity (95% CI), %	AUC (95% CI)	"True" coverage (95% CI), %	Estimated survey coverage, %	Inflation factor
No. of ANC visits						
FANC model (4 or more)	80.3 (69.1, 88.8)	51.5 (41.1, 61.4)	0.66 (0.59, 0.73)	40.8 (33.4, 48.5)	61.5	1.51
1 to 3	51.5 (41.4, 61.4)	80.3 (69.1, 88.8)	0.66 (0.59, 0.73)	59.2 (51.5, 66.6)	38.5	0.65
4	40.0 (21.1, 61.3)	14.4 (9.5, 20.5)	0.54 (0.43, 0.64)	14.4 (9.5, 20.5)	33.3	2.32
5 to 6	19.4 (7.5, 37.5)	80.4 (73.0, 86.6)	0.50 (0.42, 0.58)	17.8 (12.4, 24.3)	19.5	1.10
More than 6	20.0 (4.1, 48.1)	92.5 (87.2, 96.0)	0.56 (0.46, 0.67)	8.6 (4.9, 13.8)	8.6	1.00

[1] ANC, antenatal care; FANC, focused antenatal care.

When considering only the subgroup with complete observation (Table 2B), the binary indicator 4+ visits still had better sensitivity and AUC than the multi-categorical variable. However, the IF increased to 1.51, indicating overestimation of four or more ANC visits at the population level. Sensitivity remained low among all visit categories and had the same decreasing trend in the overall population. Specificity was relatively similar. Individual validity was still highest but not very good in the 1-3 visits category (AUC: 0.66; 95% CI: 0.59, 0.73) while others were no better than a random guess. However, two groups demonstrated great population-level validity. The 5-6 and more than 6 visits

For peer review only

groups now had IFs of 1.10 and 1.00 respectively, indicating low population bias. Overestimation still existed in the 4 visits group with an IF of 2.32.

Figure 2 is an IF graph created based on the sensitivity, specificity, and true population coverage of the binary indicator (4+ visits) among women with live births. The difference between the observed and reported coverage is illustrated by the vertical red line. As outlined in the graph, maternal report tends to overestimate the number of ANC visits at lower numbers of visits, but underestimates at higher numbers of visits. Even in subgroups with IF close to 1.00, the survey estimation could greatly deviate from the true measurement depending on the true coverage of ANC4+.

Table 3. Maternal characteristics associated with report accuracy[1]

	n (%)	Unadjusted RR (95% CI)	Adjusted RR (95% CI)
Any education	162 (40.3)	0.97 (0.75, 1.25)	0.96 (0.73, 1.24)
Any previous live birth	126 (31.3)	1.18 (0.92, 1.53)	1.22 (0.93, 1.61)
Age >=25	122 (30.4)	0.89 (0.67, 1.18)	0.92 (0.68, 1.26)
SES quartiles (ref: first)			
2	71 (17.6)	0.93 (0.65, 1.35)	0.93 (0.64, 1.34)
3	126 (31.3)	1.05 (0.79, 1.41)	0.98 (0.72, 1.31)
4	57 (14.2)	0.81 (0.52, 1.24)	0.77 (0.50, 1.21)
>1 yr since last ANC observation	76 (18.9%)	1.17 (0.87, 1.57)	0.91 (0.67, 1.24)
Number of ANC visit (ref: 1 to 3)			
4 to 7	200 (49.8)	0.70 (0.54, 0.90)[2]	0.66 (0.51, 0.87)[2]
8 or more	60 (14.9)	0.51 (0.32, 0.81)[2]	0.48 (0.30, 0.77)[2]

[1] ANC, antenatal care; SES, socioeconomic status.
[2] P < 0.05

Among the 402 women with live births, only 85 (21.1%) women's report matched exactly the ANC number as observed by the gold standard. Using categorical accuracy, 154 (38.3%) women reported correctly. The categorical accuracy rate was slightly higher in those who did not seek ANC elsewhere (41.4%) compared to those who visited other facilities (36.0%), but the difference was not statistically significant. Maternal characteristics such as education, previous birth, age, and SES were not associated

For peer review only - http://bmjopen.bmj.com/site/about/guidelines.xhtml

with reporting accuracy (Table 3). The number of total ANC visits had the strongest association with maternal report accuracy, with increasing number of ANC visits associated with lower reporting accuracy. The unadjusted risk for women who received 4-7 and 8 or more ANC visits, compared to the 1-3 visit group, was 0.70 (95% CI: 0.54, 0.90) and 0.51 (95% CI: 0.32, 0.81) respectively. After adjusting for other variables, both RR decreased slightly to 0.66 (95% CI: 0.51, 0.87) and 0.48 (95% CI: 0.30, 0.77) and remained significant. This suggested that women with 4-7 ANC visits were 34% less likely to report this information correctly during household surveys, and women who had 8 or more ANC visits were 52% less likely to recall correctly, comparing to those attended 1-3 ANC visits.

DISCUSSION

This study examined the validity of maternal report of total number of ANC visits during pregnancy in rural Nepal using data from direct ANC observation. To our knowledge, it is the first study that validates number of ANC visits as an indicator in ANC coverage measurement. In general, individual-level validity was poor among women with four or more ANC visits and moderate among women with fewer ANC visits when using categorized ANC visits as an indicator, but was higher for the binary ANC4+ indicator. The validation results of the multi-categorial variable showed that four ANC visits was often overreported. Population bias seemed to be low among women with a higher number of ANC visits, but the survey question greatly overestimates the true coverage at lower prevalence and underestimates it at higher prevalence. Less than half of women recalled the exact number of visits correctly during the postpartum visit. Reporting accuracy was found to be negatively associated with the total number of ANC visits during pregnancy but was not associated with maternal characteristics such as age, education, parity, and SES. The recall period was also not associated with accuracy of recall but there was not a wide range of recall times to examine this variable.

These validation results suggest some bias in household surveys that report number of ANC visits and that report ANC4+. At the population level, 1-3 visits were underreported, but having had four

For peer review only - http://bmjopen.bmj.com/site/about/guidelines.xhtml

ANC visits was highly overreported among the multi-categorical variable. This might be due to Nepal's guideline on ANC, which was based on the FANC model, that may have introduced bias in household surveys with women more likely to report the norm or expected number of visits for which they would be paid through the cash incentive system. Only the 1-3 visits groups showed a moderate level of individual validity, which was consistent with the regression results where more ANC visits was associated with less accurate self-report. Besides the participant's ability to recall correctly, cognitive and situational issues are usually the two factors associated with self-report validity [21]. In this case, the language used during the postpartum interview, which was specifically designed to resemble that used in the DHS, could be misunderstood. A study of cognitive testing of questions about antenatal care suggested that overreporting and underreporting may be related to the definition of an ANC visit [22]. ANC visits are meant to be regular preventive checkups in pregnancy. However, if a woman came for care because she was sick, this would be counted as an ANC visit in the gold standard observed count but might not be counted as an ANC checkup visit by the woman at the time of recall six months postpartum [22]. Social desirability bias is the inclination of people to report more socially desired activities than they actually performed (overreporting) or understate undesirable attributes (underreport) [23]. In the study scenario, women who had less than four ANC tended to report more and meet the social standard in front of the interviewer and sometimes the presence of their husbands, resulting in the underreporting of 1-3 visits group and overreporting of the 4 visits group. The low population bias here may be explained by the low prevalence of 5-6 visits due to a low number of false negatives. People who had more than six ANC visits seemed to underreport their receipt of care. This might be attributed to the respondents' inability to recall higher number of visits. ANC visits are often concentrated towards the end of pregnancy. Women might have conflated the visits in their minds and recalled a lower number. A study of social desirability bias was undertaken as part of this validity study. It showed very little social desirability bias but did show situational bias associated with whether family

For peer review only - http://bmjopen.bmj.com/site/about/guidelines.xhtml

members or others were present during the postpartum interview (Thorne-Lyman, unpublished data, 2023). It was found that the presence of any adult at the interview is associated with greater risk of overestimation of ANC frequency, with the presence of the husband being the most influential (Thorne-Lyman, unpublished data, 2023).

There have been several yet limited studies on the validity of health indicators in coverage measurement. This paper contributes to the current body of validation studies and factors related to the accuracy of ANC self-report. One similar study evaluated the coverage rate of intermittent preventive treatment during pregnancy based on mother's recall in Benin, Ghana, Malawi, and Tanzania [24]. It was found that compared to ANC card data (the gold standard), recalled data in household surveys were valid [24]. Sensitivity and specificity of self-report were generally higher than that in our study, and notably, the AUC of reported measurements from all four countries was higher than 0.8 [24]. One potential reason for the different conclusions between the two studies could be that the recommended frequency for intermittent preventive treatment (at least 3 times) is lower than that of ANC, resulting in less variation in the total number of ANC visits and making it easier to recall correctly during household surveys. Additionally, ANC cards were used as the gold standard in their case instead of health facility records [24]. This could bias the results as women's self-reported validity might be associated with their ability to keep health records, which makes ANC cards not an optimal source for verification. Those who had a card would be more likely to read the card and be reminded of the number of visits they had.

In this study, the binary indicator 4+ visits performed better than multi-categorized indicators (1-3 visits, 4 visits, 5-6 visits, and more than 6 visits) in terms of both individual- and population-level validity. In previous studies, dichotomous indicators often possessed higher validity than counts or frequency for the same intervention in household surveys. For example, the NNIPS study on iron-folic acid found that report of "any iron-folic acid receipt" demonstrated better individual validity and very low population bias compared to specific tablet counts [17]. However, in this study, the prevalence of

For peer review only

receipt of any iron-folic acid was very high (over 95%), which is likely the primary reason for higher validity and low bias. Furthermore, in a study comparing national household survey and health facility service statistics in Uganda, there was considerable agreement between the two data sources for skilled attendance at birth and at least four ANC visits [25]. However, if the number of ANC visits were dichotomized at eight times, the validity might not be better than that of categorical indicators. Many studies also have found that report accuracy was associated with the length of recall period, where accuracy decreases with extended duration of recall [26-28], but such relation was not seen in the NNIPS studies.

A strength of this study is that the gold standard used in validation was through direct observation by trained field workers. Study observers were all trained to reach a standard level of validity before working at the study sites, which makes it a more objective and reliable source for verification than secondary databases. A second strength is that the study had a reasonable length of recall period, not as long as DHS but longer than many other studies. Other validation studies use recall periods of less than six months or even exit interviews to validate maternal report. One of the main limitations is that the study only considered women who presented for their first ANC at public health posts. Women who never attend ANC or those who do not go to public facilities were not captured through the study, but they may have characteristics that influence the overall self-report validity. Another limitation is that the study was unable to observe women if they went to other facilities for ANC. Subgroup analysis with just those women with all their ANC visits observed was conducted for more rigorous validation results. However, these measures were dependent on the women's ability to recall and report their care-seeking behavior at other clinics. Lastly, the study was limited to only five health posts across two municipalities. Thus, the study result may not be generalizable to all women in the 20 Sarlahi municipalities, or Nepal's rural population in general.

CONCLUSION

For peer review only - http://bmjopen.bmj.com/site/about/guidelines.xhtml

In general, the number of ANC visits as asked during DHS or household surveys, was not accurately recalled, although ANC4+ (a major marker of ANC coverage progress) recalled better than if ANC was more finely categorized. For women with more ANC visits than the standard of 4 (for Nepal at the time of this study), women tended to underreport the number of ANC visits. With the change from 4 or more to 8 or more ANC visits as the standard, approaches to improving recall should be identified and implemented.

For peer review only

Author Contributions: MM and JK designed the study. TPL, SKK, SLC, JK and EB contributed to the implementation of the study. XX conducted the analyses and wrote the first draft of the manuscript. JK submitted edits to the manuscript. MM, JK, TPL, SKK, EB and SCL all have read and approved the final version of the manuscript. JK acted as the guarantor.

Funding: This research was funded by the Bill and Melinda Gates Foundation (grant number OPP1172551).

Competing interests: None declared.

Patient consent for publication: Not applicable.

Ethics approval: The Institutional Review Board of the Johns Hopkins Bloomberg School of Public Health and the Nepal Health Research Council approved the parent study.

Data availability statement: Data are available upon reasonable request.

For peer review only

REFERENCES

1. World Health Organization. Trends in maternal mortality 2000 to 2017: Estimates by WHO, UNICEF, UNFPA, World Bank Group and the United Nations Population Division. Geneva: WHO, 2019.
2. UNICEF DATA. Neonatal mortality [online]. January 2023. https://data.unicef.org/topic/child-survival/neonatal-mortality/ (accessed 20 April 2023).
3. World Health Organization. Maternal mortality [online]. February 2023. https://www.who.int/news-room/fact-sheets/detail/maternal-mortality (accessed 20 April 2023).
4. Wondemagegn AT, Alebel A, Tesema C, Abie W. The effect of antenatal care follow-up on Neonatal Health Outcomes: A systematic review and meta-analysis. Public Health Reviews. 2018;39(1). doi:10.1186/s40985-018-0110-y
5. Tekelab T, Chojenta C, Smith R, Loxton D. The impact of antenatal care on neonatal mortality in sub-Saharan africa: A systematic review and meta-analysis. PLOS ONE. 2019;14(9). doi:10.1371/journal.pone.0222566
6. World Health Organization. WHO recommendations on antenatal care for a positive pregnancy experience. World Health Organization. Geneva: WHO, 2016.
7. Mchenga M, Burger R, von Fintel D. Examining the impact of who's focused antenatal care policy on early access, underutilisation and quality of antenatal care services in Malawi: A retrospective study. BMC Health Services Research. 2019;19(1). doi:10.1186/s12913-019-4130-1
8. McNellan CR, Dansereau E, Wallace MC, et al. Antenatal care as a means to increase participation in the continuum of maternal and Child healthcare: An analysis of the poorest regions of four Mesoamérican countries. BMC Pregnancy and Childbirth. 2019;19(1). doi:10.1186/s12884-019-2207-9
9. Aryal KK, Sharma SK, Khanal MN, et al. Maternal health care in Nepal: Trends and determinants (English). DHS Further Analysis Reports No. 118. The DHS Program, 2019.
10. Khatri RB, Durham J, Assefa Y. Utilisation of quality antenatal, delivery and postnatal care services in Nepal: An Analysis of Service Provision Assessment. Globalization and Health. 2021;17(1). doi:10.1186/s12992-021-00752-x
11. Powell-Jackson T, Neupane BD, Tiwari S, et al. The impact of Nepal's National Incentive Programme to promote safe delivery in the district of Makwanpur. Innovations in Health System Finance in Developing and Transitional Economies. 2009;221–49. doi:10.1108/s0731-2199(2009)0000021012
12. Ensor T, Bhatt H, Tiwari S. Incentivizing Universal Safe Delivery in Nepal: 10 years of experience. Health Policy and Planning. 2017;32(8):1185–92. doi:10.1093/heapol/czx070
13. Ministry of Health and Population. National Medical Standard for maternal and newborn care volume III. Nepal: Ministry of Health and Population, 2020.
14. Ministry of Health. Nepal Demographic and Health Survey 2016. Kathmandu, Nepal: Ministry of Health, Nepal, 2017.
15. Bryce E, Katz J, Pema Lama T, et al. Antenatal care processes in rural southern Nepal: Gaps in and quality of service provision—a cohort study. BMJ Open. 2021;11(12). doi:10.1136/bmjopen-2021-056392
16. Bryce E, Katz J, Heidkamp R, et al. Validation of maternal report of nutrition-related interventions and counselling during antenatal care in southern Nepal. Maternal & Child Nutrition. 2021;18(2). doi:10.1111/mcn.13303
17. Bryce E, Munos M, Lama TP, et al. Validation of maternal report of receipt of iron–folic acid supplementation during antenatal care in rural southern Nepal. The Journal of Nutrition. 2022;152(1):310–8. doi:10.1093/jn/nxab336
18. Central Bureau of Statistics, Government of Nepal. National Population and Housing Census 2011. Kathmandu, Nepal: Central Bureau of Statistics, Government of Nepal, 2012.

For peer review only - http://bmjopen.bmj.com/site/about/guidelines.xhtml

19. Wee DW, Mullany LC, Katz J, et al. Pregnancy-related mortality in southern Nepal between 2001 and 2006: Independent estimates from a prospective, population-based cohort and a direct Sisterhood survey. American Journal of Epidemiology. 2010;172(7):855–60. doi:10.1093/aje/kwq207
20. Munos MK, Blanc AK, Carter ED, et al. Validation studies for population-based intervention coverage indicators: Design, analysis, and interpretation. Journal of Global Health. 2018;8(2). doi:10.7189/jogh.08.020804
21. Brener ND, Billy JOG, Grady WR. Assessment of factors affecting the validity of self-reported health-risk behavior among adolescents: Evidence from the scientific literature. Journal of Adolescent Health. 2003;33(6):436–57. doi:10.1016/s1054-139x(03)00052-1
22. L-Thorne-Lyman A, Lama TP, Heidkamp RA, et al. Cognitive testing of questions about antenatal care and nutrition interventions in southern Nepal. Social Science & Medicine. 2022;311:115318. doi:10.1016/j.socscimed.2022.115318
23. Latkin CA, Edwards C, Davey-Rothwell MA, et al. The relationship between social desirability bias and self-reports of health, substance use, and social network factors among urban substance users in Baltimore, Maryland. Addictive Behaviors. 2017;73:133–6. doi:10.1016/j.addbeh.2017.05.005
24. Hansen N, Youll S, Florey L, Taylor C. Intermittent preventive treatment of malaria in pregnancy coverage estimates from population-based surveys: Reliability of Women's recall among women with ANC cards. The American Journal of Tropical Medicine and Hygiene. 2021;105(2):472–6. doi:10.4269/ajtmh.20-1296
25. Simmons EM, Singh K, Mpiima J, et al. Assessing coverage of essential maternal and child health interventions using health-facility data in Uganda. Population Health Metrics. 2020;18(1). doi:10.1186/s12963-020-00236-x
26. Zimmerman LA, Shiferaw S, Seme A, et al. Evaluating consistency of recall of maternal and newborn care complications and intervention coverage using PMA panel data in SNNPR, Ethiopia. PLOS ONE. 2019;14(5). doi:10.1371/journal.pone.0216612
27. D'Souza-Vazirani D, Minkovitz CS, Strobino DM. Validity of maternal report of acute health care use for children younger than 3 years. Archives of Pediatrics & Adolescent Medicine. 2005;159(2). doi:10.1001/archpedi.159.2.167
28. Kanyangarara M, Katz J, Munos MK, et al. Validity of self-reported receipt of iron supplements during pregnancy: Implications for coverage measurement. BMC Pregnancy and Childbirth. 2019;19(1). doi:10.1186/s12884-019-2247-1

For peer review only

For peer review only - http://bmjopen.bmj.com/site/about/guidelines.xhtml

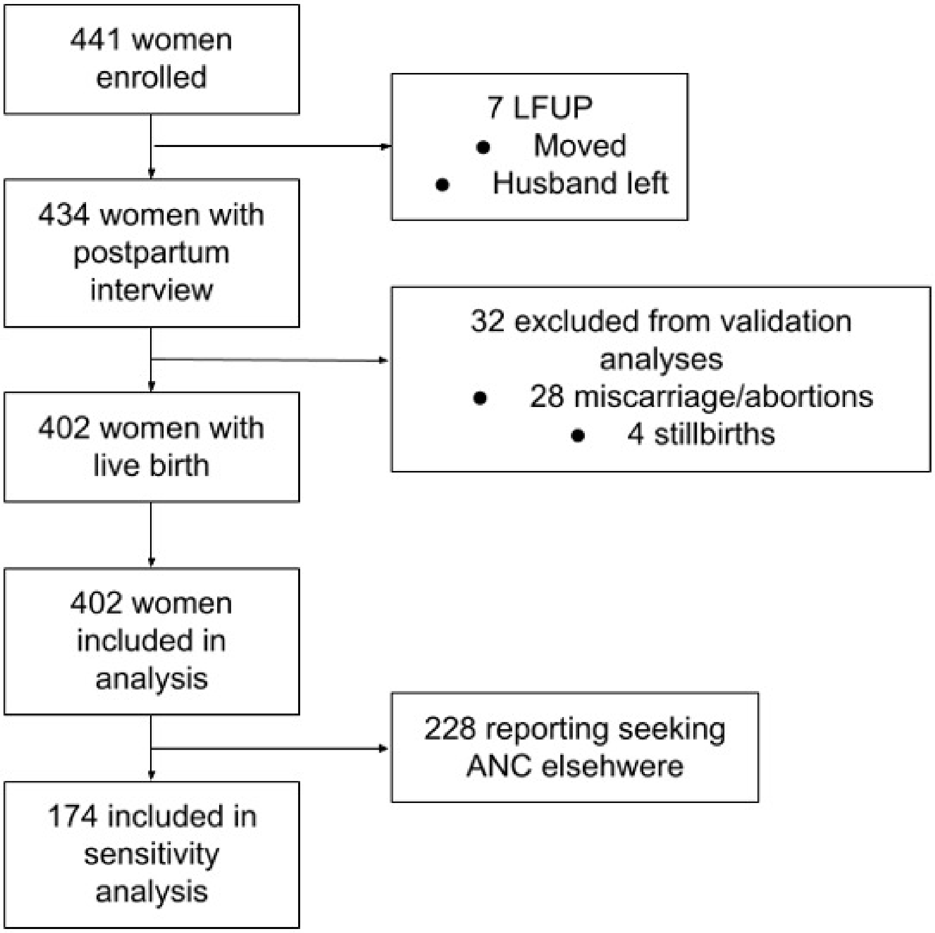

Figure 1. Participant flowchart. ANC, antenatal care; LFUP, lost to follow-up.

102x102mm (144 x 144 DPI)

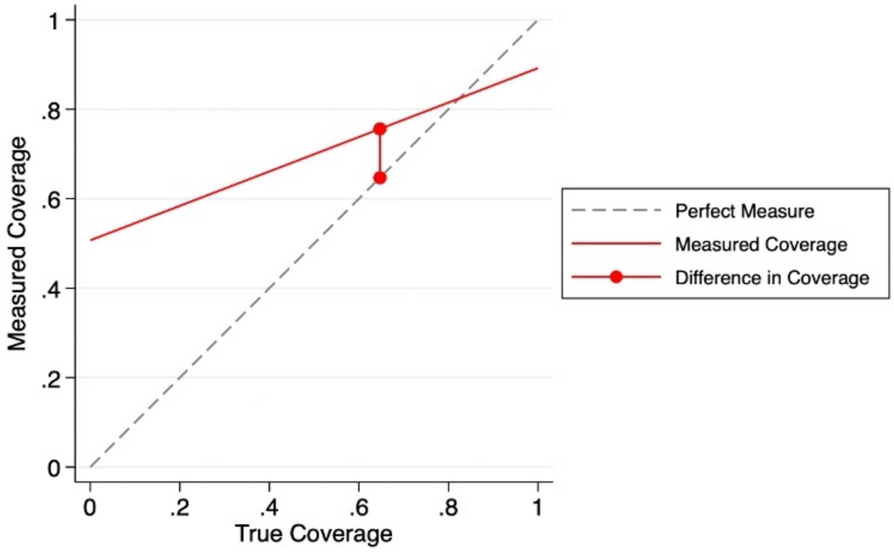

Figure 2. Ture coverage compared with measured coverage for four or more ANC visits.

147x98mm (144 x 144 DPI)

SUPPLEMENTAL TABLES AND FIGURES

Supplemental Table 1. Characteristics of participants with postpartum interview

Characteristic	Observed all ANC visits (N=204)		Received ANC between observations (N=230)		Two sample t-test p-value	Total (N=434)	
	Mean (SD)	Range	Mean (SD)	Range		Mean (SD)	Range
Woman's age, years	22.8 (4.3)	16 to 41	22.3 (4.0)	16 to 35	0.184	22.5 (4.2)	16 to 41
Total number of ANC visits observed	3.2 (2.0)	1 to 10	5.6 (2.3)	2 to 14	<0.01	4.5 (2.5)	1 to 14
Number of months between last ANC observation and postpartum interview	11.6 (3.3)	3 to 22	9.1 (2.5)	3 to 17	<0.01	10.3 (3.2)	3 to 22

	Observed all ANC visits (N=204)		Received ANC between observations (N=230)		Chi-square p-value	Total (N=434)	
Most recent pregnancy outcome							
Miscarriage/abortion	27	13.2%	1	0.4%	<0.01	28	6.5%
Stillbirth	3	1.5%	1	0.4%		4	0.9%
At least one live birth	174	85.3%	228	99.1%		402	92.6%
4 quantiles of SES							
1	94	46.1%	73	31.7%	<0.01	167	38.5%
2	39	19.1%	35	15.2%		74	17.1%
3	48	23.5%	84	36.5%		132	30.4%
4	23	11.3%	38	16.5%		61	14.1%
Is this the woman's first pregnancy?							
No	153	75.0%	145	63.0%	<0.01	298	68.7%
Yes	51	25.0%	85	37.0%		136	31.3%
Did the woman receive any years of education?							
No	139	68.1%	120	52.2%	<0.01	259	59.7%
Yes	65	31.9%	110	47.8%		175	40.3%
Trimester at enrollment							
1-3 months	81	39.7%	109	47.4%	0.1	190	43.8%
4-6 months	117	57.4%	119	51.7%		236	54.4%
7-9 months	6	2.9%	2	0.9%		8	1.8%

For peer review only

Supplemental Figure 1. Agreement between numbers of ANC visits observed at health posts and maternal report at postpartum interview in (A) the entire cohort (n = 402) and (B) the subgroup with all ANC observed (n = 174).

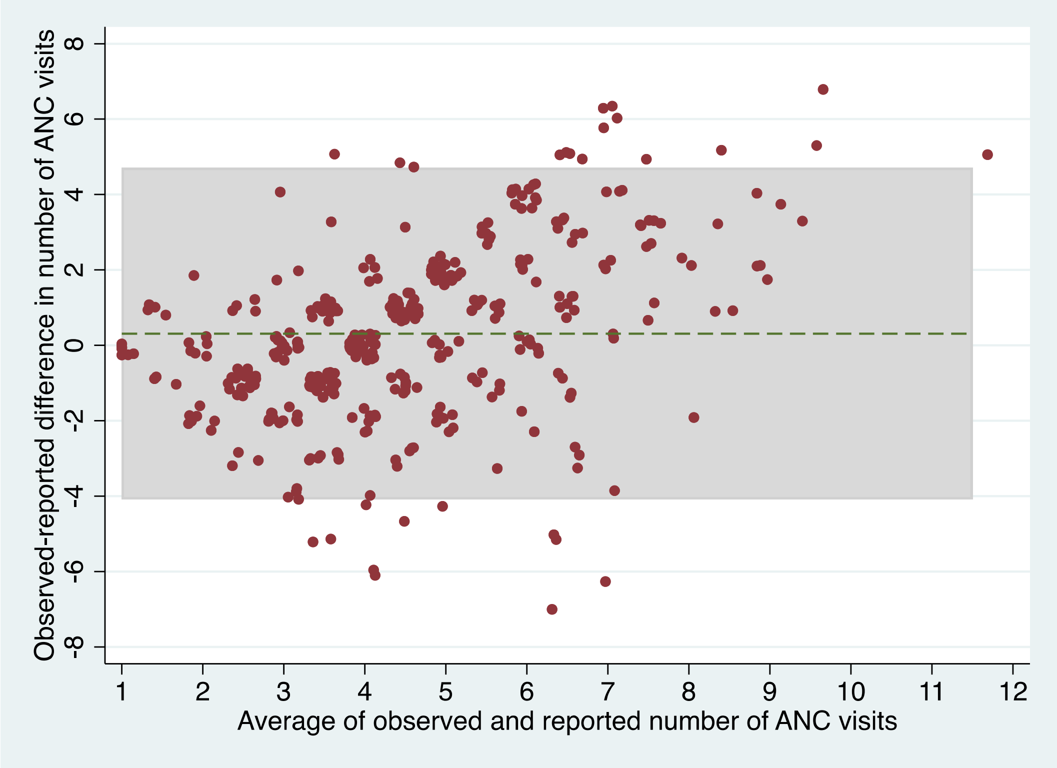

(A)

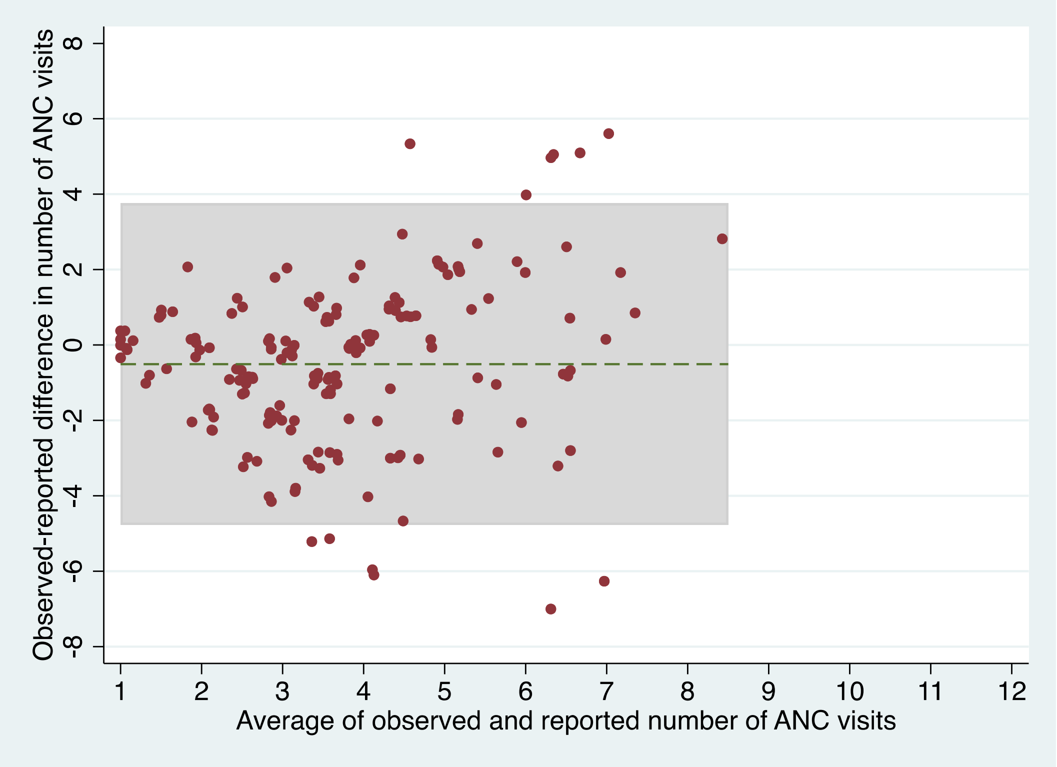

(B)

Supplemental Figure 2. Histogram of observed number of ANC visits (n = 402).

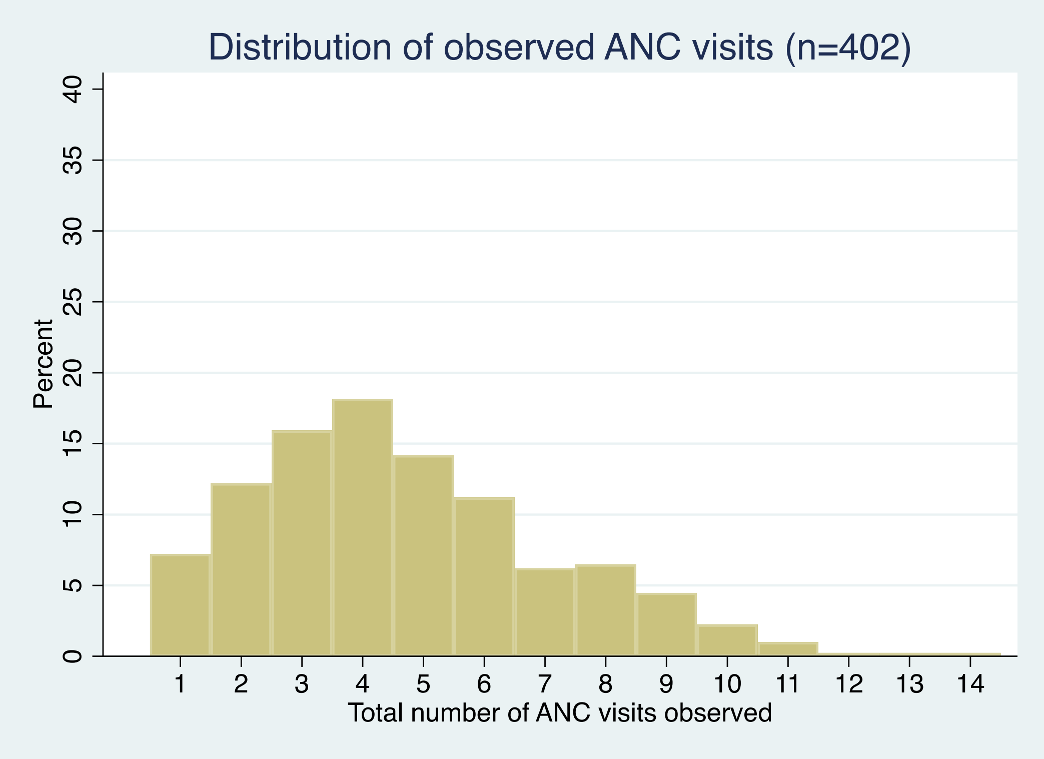

Supplemental Figure 3. Histogram of reported number of ANC visits (n = 402).

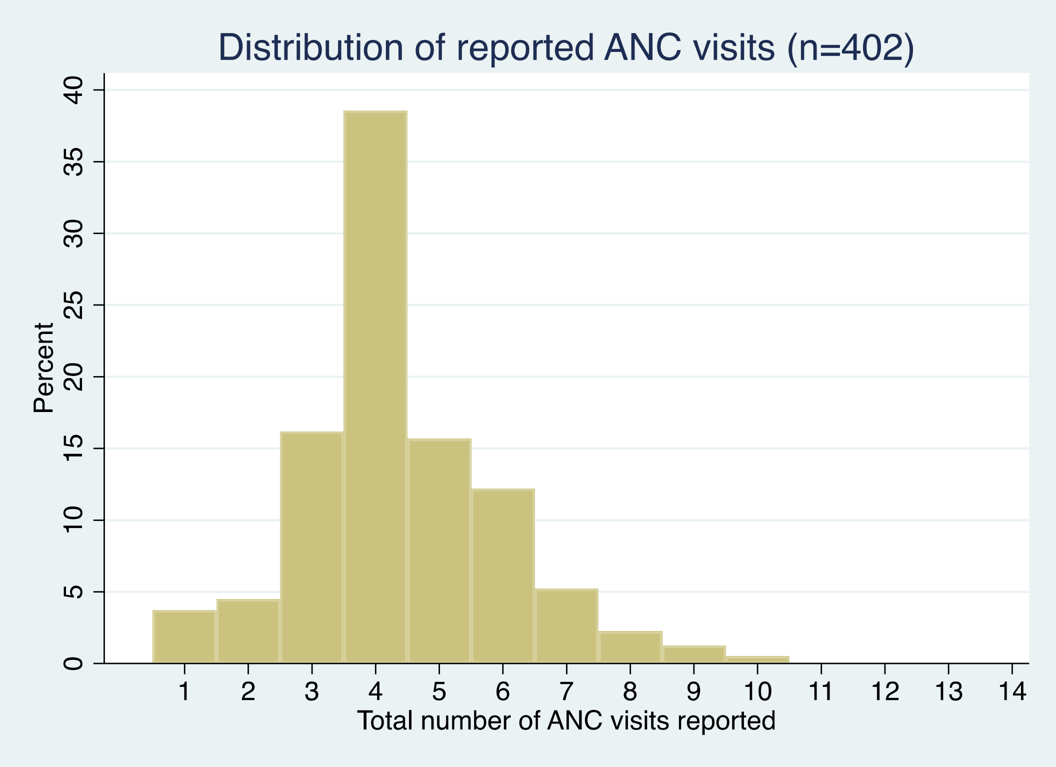

STROBE Statement—Checklist of items that should be included in reports of ***cohort studies***

	Item No	Recommendation	Page No
**Title and abstract**	1	(*a*) Indicate the study's design with a commonly used term in the title or the abstract<br>(*b*) Provide in the abstract an informative and balanced summary of what was done and what was found	1-2
**Introduction**			
Background/rationale	2	Explain the scientific background and rationale for the investigation being reported	4
Objectives	3	State specific objectives, including any prespecified hypotheses	5
**Methods**			
Study design	4	Present key elements of study design early in the paper	6
Setting	5	Describe the setting, locations, and relevant dates, including periods of recruitment, exposure, follow-up, and data collection	5
Participants	6	(*a*) Give the eligibility criteria, and the sources and methods of selection of participants. Describe methods of follow-up<br>(*b*) For matched studies, give matching criteria and number of exposed and unexposed	6
Variables	7	Clearly define all outcomes, exposures, predictors, potential confounders, and effect modifiers. Give diagnostic criteria, if applicable	9
Data sources/ measurement	8*	For each variable of interest, give sources of data and details of methods of assessment (measurement). Describe comparability of assessment methods if there is more than one group	6
Bias	9	Describe any efforts to address potential sources of bias	9
Study size	10	Explain how the study size was arrived at	7
Quantitative variables	11	Explain how quantitative variables were handled in the analyses. If applicable, describe which groupings were chosen and why	9
Statistical methods	12	(*a*) Describe all statistical methods, including those used to control for confounding<br>(*b*) Describe any methods used to examine subgroups and interactions<br>(*c*) Explain how missing data were addressed<br>(*d*) If applicable, explain how loss to follow-up was addressed<br>(*e*) Describe any sensitivity analyses	9
**Results**			
Participants	13*	(a) Report numbers of individuals at each stage of study—eg numbers potentially eligible, examined for eligibility, confirmed eligible, included in the study, completing follow-up, and analysed<br>(b) Give reasons for non-participation at each stage<br>(c) Consider use of a flow diagram	9
Descriptive data	14*	(a) Give characteristics of study participants (eg demographic, clinical, social) and information on exposures and potential confounders<br>(b) Indicate number of participants with missing data for each variable of interest<br>(c) Summarise follow-up time (eg, average and total amount)	11
Outcome data	15*	Report numbers of outcome events or summary measures over time	12

For peer review only

For peer review only - http://bmjopen.bmj.com/site/about/guidelines.xhtml

Main results	16	(*a*) Give unadjusted estimates and, if applicable, confounder-adjusted estimates and their precision (eg, 95% confidence interval). Make clear which confounders were adjusted for and why they were included<br>(*b*) Report category boundaries when continuous variables were categorized<br>(*c*) If relevant, consider translating estimates of relative risk into absolute risk for a meaningful time period	14
Other analyses	17	Report other analyses done—eg analyses of subgroups and interactions, and sensitivity analyses	14
**Discussion**			
Key results	18	Summarise key results with reference to study objectives	15
Limitations	19	Discuss limitations of the study, taking into account sources of potential bias or imprecision. Discuss both direction and magnitude of any potential bias	18
Interpretation	20	Give a cautious overall interpretation of results considering objectives, limitations, multiplicity of analyses, results from similar studies, and other relevant evidence	15
Generalisability	21	Discuss the generalisability (external validity) of the study results	18
**Other information**			
Funding	22	Give the source of funding and the role of the funders for the present study and, if applicable, for the original study on which the present article is based	19

*Give information separately for exposed and unexposed groups.

Note: An Explanation and Elaboration article discusses each checklist item and gives methodological background and published examples of transparent reporting. The STROBE checklist is best used in conjunction with this article (freely available on the Web sites of PLoS Medicine at http://www.plosmedicine.org/, Annals of Internal Medicine at http://www.annals.org/, and Epidemiology at http://www.epidem.com/). Information on the STROBE Initiative is available at http://www.strobe-statement.org.

For peer review only

BMJ Open

Validation of maternal recall of number of antenatal care visits attended in rural Southern Nepal: a longitudinal cohort study

Journal:	*BMJ Open*
Manuscript ID	bmjopen-2023-079029.R1
Article Type:	Original research
Date Submitted by the Author:	30-Oct-2023
Complete List of Authors:	Xie, Xinyu; Johns Hopkins University Bloomberg School of Public Health, International Health<br>Munos, Melinda; Johns Hopkins University, Bloomberg School of Public Health, Department of International Health<br>Plama, Tsering; Johns Hopkins University Bloomberg School of Public Health, Department of International Health<br>Bryce, Emily; Johns Hopkins University Bloomberg School of Public Health,<br>Khatry, Subarna; Nepal Nutrition Intervention Project - Sarlahi<br>LeClerq, Steven C.; Johns Hopkins Bloomberg School of Public Health, Department of International Health; Nepal Nutrition Intervention Project<br>Katz, Joanne; Johns Hopkins University,
**Primary Subject Heading**:	Global health
Secondary Subject Heading:	Public health
Keywords:	Quality in health care < HEALTH SERVICES ADMINISTRATION & MANAGEMENT, Patient Reported Outcome Measures, Surveys and Questionnaires

SCHOLARONE™
Manuscripts

I, the Submitting Author has the right to grant and does grant on behalf of all authors of the Work (as defined in the below author licence), an exclusive licence and/or a non-exclusive licence for contributions from authors who are: i) UK Crown employees; ii) where BMJ has agreed a CC-BY licence shall apply, and/or iii) in accordance with the terms applicable for US Federal Government officers or employees acting as part of their official duties; on a worldwide, perpetual, irrevocable, royalty-free basis to BMJ Publishing Group Ltd ("BMJ") its licensees and where the relevant Journal is co-owned by BMJ to the co-owners of the Journal, to publish the Work in this journal and any other BMJ products and to exploit all rights, as set out in our licence.

The Submitting Author accepts and understands that any supply made under these terms is made by BMJ to the Submitting Author unless you are acting as an employee on behalf of your employer or a postgraduate student of an affiliated institution which is paying any applicable article publishing charge ("APC") for Open Access articles. Where the Submitting Author wishes to make the Work available on an Open Access basis (and intends to pay the relevant APC), the terms of reuse of such Open Access shall be governed by a Creative Commons licence – details of these licences and which Creative Commons licence will apply to this Work are set out in our licence referred to above.

Other than as permitted in any relevant BMJ Author's Self Archiving Policies, I confirm this Work has not been accepted for publication elsewhere, is not being considered for publication elsewhere and does not duplicate material already published. I confirm all authors consent to publication of this Work and authorise the granting of this licence.

For peer review only

Title:

Validation of maternal recall of number of antenatal care visits attended in rural Southern Nepal: a longitudinal cohort study

Authors and Institutions: Xinyu Xie, Melinda Munos, Tsering Pema Lama, Emily Bryce, Subarna Khatry, Steven LeClerq, Joanne Katz

Keywords: Nepal, antenatal care, recall, validity, coverage, measurement

Correspondence to: Xinyu Xie; 615 N. Wolfe Street, Baltimore, MD 21205; evaxie.shchn@gmail.com, +1 443-839-5016

For peer review only

ABSTRACT

Objectives: This study aimed to examine the validity of maternal recall of total number of antenatal care (ANC) visits during pregnancy and factors associated with the accuracy of maternal recall.

Design: This was a longitudinal cohort study conducted from December 2018 through November 2020.

Setting: Five government health posts in the Sarlahi District of Southern Nepal

Participants: 402 pregnant women between ages of 15 and 49 who presented for their first ANC visit at the study health posts

Main Outcomes: The observed number of ANC visits (gold standard) and the reported number of ANC visits at the postpartum interview (maternal recall)

Results: On average, women in the study who had a live birth attended 4.7 ANC visits. About 65% of them attended four or more ANC visits during pregnancy as recommended by the Nepal government, and 38.3% of maternal report matched the categorical ANC visits as observed by the gold standard. The individual validity was poor to moderate, with the highest AUC being 0.69 (95% CI: 0.65, 0.74) in the 1-3 visits group. Population-level bias (as distinct from individual-level bias) was observed in the 1-3 visits and 4 visits groups, where 1-3 visits were underreported (IF: 0.69) and 4 ANC visits were highly overreported (IF: 2.12). The binary indicator ANC4+ (1-3 visits versus 4+ visits) showed better population-level validity (AUC: 0.69; IF: 1.17) compared with the categorical indicators (1-3 visits, 4 visits, 5-6 visits, and more than 6 visits). Report accuracy was not associated with maternal characteristics but was related to ANC frequency. Women who attended more ANC visits were less likely to correctly report their total number of visits.

Conclusion: Maternal report of number of ANC visits during pregnancy may not be a valid indicator for measuring ANC coverage. Improvements are needed to measure the frequency of ANC visits.

For peer review only - http://bmjopen.bmj.com/site/about/guidelines.xhtml

ARTICLE SUMMARY

Strengths and limitations of this study

- The gold standard was established using direct observation by trained field workers, thus eliminating the risk of recall bias.
- The study observers were all trained to reach a standard level of validity before working at the study sites, which provides a more objective and reliable source for verification than secondary databases.
- The study had an appropriate length of recall period comparing to other validation studies who use recall periods of less than six months or even exit interviews to validate maternal report.
- The study only considered women who presented for their first ANC at public health posts for the feasibility of data collection.
- Women who visited facilities other than the study health posts were not observed but were asked to recall how many other ANC visits they attended.

For peer review only

INTRODUCTION

The United Nations Inter-Agency Group estimated that in 2020 the global maternal mortality ratio was 223 deaths per 100,000 live births, and UNICEF reported an average global neonatal mortality rate of 18 deaths per 1,000 live births in 2021 [1,2]. Maternal and neonatal mortality remains an issue that differentially impacts developed and developing countries. According to the World Health Organization (WHO), 94% of all maternal deaths in 2017 occurred in low- and middle-income countries, with 86% taking place in sub-Saharan Africa and South Asia [3]. Sub-Saharan Africa and South Asia also have the highest neonatal mortality rate among all regions (27 and 23 deaths per 1,000 live births respectively in 2021) [2].

Antenatal care (ANC) plays an important role in maternal and neonatal health. By providing health contacts with the mother at key points in the continuum of care, quality ANC greatly reduces the risk of maternal mortality through preventive and promotive care and early detection and treatment of pregnancy-related complications, improving the survival and health of newborns [3-5]. In 2002, the WHO introduced the focused ANC (FANC) model consisting of at least four ANC visits during pregnancy.

The Ministry of Health and Population (MoHP) in Nepal followed the FANC model with at least four ANC visits at the 4th, 6th, 8th, and 9th month of gestation when they conducted the Nepal Demographic and Health Survey (DHS) in 2016 [6]. To improve the utilization of ANC, the Nepal government started a national Safe Delivery Incentive Program, or Aama Program in Nepali [7]. This program provides monetary incentives to women who completed at least four ANC visits as suggested by the MoHP and women who delivered at health facilities by skilled birth attendants [7]. However, studies have found that recipients of the incentives were disproportionally wealthy families that had more access to health services and policy information, and the program had limited effect on ANC utilization in rural areas [8,9]. The MoHP published the National Medical Standard for Maternal and

For peer review only - http://bmjopen.bmj.com/site/about/guidelines.xhtml

Newborn Care in 2020, stating that Nepal now recommends the new WHO eight contacts of ANC approach [10].

According to the 2022 Nepal DHS, the ANC service utilization rate was 94% for at least one ANC visit among 15- to 49-year-old women who had a live or stillbirth within two years before the survey; 80% of women had four or more ANC visits during their latest pregnancy and 82% of women in rural regions had at least four ANC visits [11].

Household surveys like DHS and the Multiple Indicator Cluster Survey Program (MICS) have been primary data sources for national level health statistics across the world and will continue to be a major tool for routine tracking of coverage and quality of care in developing countries. Nepal has a national household survey every five years to evaluate the national ANC coverage, and the frequency of ANC visits serves as an important indicator. However, the survey often takes place many years after a woman's pregnancy. It is unknown whether the woman can correctly recall the total number of ANC visits and provide accurate answers to the DHS question. Therefore, the validity of this question in such household surveys is unknown. Previous studies have investigated the validity of ANC coverage indicators like quality of care, nutritional interventions, nutrition counselling, and iron-folic acid supplementation in the same Nepal cohort, but the validity of frequency of ANC visits has not been explored [12-14]. The objective of this study is to examine the validity of maternal report of total number of ANC visits and factors associated with the accuracy of maternal report.

METHODS

Study site

This longitudinal cohort study was conducted from December 2018 to November 2020 within the study area of the Nepal Nutrition Intervention Project Sarlahi (NNIPS) located in the rural Sarlahi District of Southern Nepal. Sarlahi is a part of the Madhesh Province bordered to the west by the Bagmati River and to the south by the state of Bihar, India. Two municipalities (Haripur and Kabilasi)

For peer review only - http://bmjopen.bmj.com/site/about/guidelines.xhtml

were chosen based on the census data and experiences from the local study team. Sarlahi district has a female population of 379,973 and 47.8% of these are between 15 to 49 years of age [15]. Previous studies in the NNIPS area showed that women in Sarlahi district had an estimated pregnancy-related mortality ratio of 529 deaths per 100,000 live births in the period 2001-2006, which was almost twice of the national average [16]. Nepal DHS does not report maternal mortality ratios at the district level so there is no more recent comparable data. Approximately 60% of women in this area attended four or more ANC visits in the period 2010-2016, which is lower than the average among rural regions [12]. Most ANC, especially in rural areas, is provided through public facilities, although there are some private facilities and hospitals. Five public health posts at Pharadwa, Laxmipur, Pidari, Pipariya, and Kabilasi village development committees (VDCs) were designated to be the study sites because of their high attendance at ANC and accessibility to both the clients and the study team. VDCs have now been dissolved, but at the time of the study, VDCs were the smallest administrative unit in district where each VDC had nine wards.

Study population, design, and data collection

All pregnant women aged 15 years and older who lived in the NNIPS area who came for their first ANC visit, regardless of gestational age at this visit, to one of the five study health posts were eligible for the study. Women in the study were assumed to be married since it would be culturally inappropriate to ask about their marital status if they were pregnant and seeking ANC. Women who were younger than 15 years old were not enrolled. Women were considered ineligible to participate if they had already attended ANC or an ultrasound appointment before recruitment because not all ANC visits would be observed by the study team. Those who planned on visiting other health facilities than the five study ones for ANC during pregnancy were also considered ineligible for the same reason. Women who planned on leaving the NNIPS area during the study period, or up until six months after delivery were excluded to prevent and minimize any loss to follow-up. Participants were consented at

For peer review only - http://bmjopen.bmj.com/site/about/guidelines.xhtml

the enrollment visit and during the postpartum interview respectively. All women signed consent with a witness signature for those who were illiterate. Married women 15-17 living with their husbands are considered emancipated minors in Nepal and the local institutional review board approved that they could consent for themselves.

The overall study approach is to assess the validity of maternal report by comparing the observed number of ANC visits (gold standard) to the answers provided by women in the six-month postpartum interview. Trained field workers were present all day during regular hours (10 am to 4 pm) at the health posts. This was done to be able to observe all participant return visits for ANC to create the gold standard against which to compare maternal recall of number of visits. During the enrollment period, trained field workers collected the demographic data of eligible participants, such as women's age, gestational age, parity and education level. Once enrolled, the participants were asked to complete a follow-up survey at each of their ANC visits. Trained field workers recorded their presence at the ANC visit and asked them questions about any health-seeking behavior since the last visit. The follow-up form asked questions like "what is the location of your most recent ANC visit" to help determine if the woman attended any ANC that was not observed by the study team. These direct observations served as the "gold standard" for the validation analysis. A postpartum interview was conducted approximately six months after the woman's delivery to collect information on the ANC services they received during pregnancy. Some of the interview questions were constructed using the same language as the 2016 Nepal DHS. Specifically, the question about the number of ANC visits attended in the most recent pregnancy was identical to the question in the Nepal DHS ("How many times did you receive antenatal care during this pregnancy?"). The exact Nepali used in the Nepal DHS was used for this question. The interview also collected information on their socioeconomic status (SES) through questions about housing, household asset ownership, cooking fuels, and ownership of land and household goods.

Analysis

For peer review only - http://bmjopen.bmj.com/site/about/guidelines.xhtml

The study aimed to enroll 450 women to reach a sample size of 300, to estimate validation measures with sufficient precision (with prevalence of 50%, a 95% confidence interval would be 13% wide or +/- 6.5% points), accounting for women who did not have a live birth, those who may have gone elsewhere for some ANC visits and did not have all visits observed, and loss to follow-up. Eventually 441 women were enrolled in the study and 434 of them participated in the postpartum interview.

The gold standard of observed number of visits was compared to the maternal report of the number of ANC visits for the validity analysis. Since it was impractical to follow women everywhere throughout their pregnancy, the follow-up survey at each ANC visit collected information to determine whether women received ANC at facilities other than the five designated health posts where observers were stationed. Participants were categorized into those who sought ANC elsewhere and those for whom all ANC was observed by the study team. In this way, a stricter gold standard was available for subgroup analysis.

The study cohort was categorized by the total number of ANC visits: 1-3 versus 4 or more (4+) visits; 1-3 visits, 4 visits, 5-6 visits, and more than 6 visits. Since the Nepal MoHP recommended four or more ANC visits during pregnancy at the time of the study, the 4+ visits group was designed to see the compliance of FANC model and test the validity of a binary ANC frequency indicator. Individual validity was evaluated through sensitivity, specificity, and area under the receiver operating characteristic curve (AUC). To calculate sensitivity and specificity, 2x2 tables were constructed. Each participant was assigned to a cell in the table based on whether their ANC visit number fell in the group according to the gold standard and the maternal report. The calculation of sensitivity and specificity is similar to that of a diagnostic test. AUC in this scenario represents the probability that a woman's report of number of ANC visits is consistent with the gold standard category. AUC is calculated as the area under the plot of sensitivity vs. (1-specificity) [17]. An AUC higher than 0.7 is considered as high individual-level accuracy; an AUC of 0.5 indicates that maternal report on the indicator is no better than a random guess [17].

For peer review only

For peer review only - http://bmjopen.bmj.com/site/about/guidelines.xhtml

Population-level validity was measured through the inflation factor (IF), which gives an estimate of the accuracy of the postpartum survey in reflecting the true coverage in the population. It is calculated as the study coverage measured from maternal report divided by the true population coverage value based on the gold standard. The study coverage can be calculated using the formula: Pr=P(SN+SP-1)+(1-SP), where Pr is the study coverage, P is the true population coverage, SN is sensitivity, and SP is specificity [17]. An IF of 1.00 indicates perfect accuracy and an IF between 0.75 and 1.25 means there is low population-level bias [17].

Bivariate and multivariate log-binomial regression models were used to assess factors associated with accuracy of maternal report. The primary outcome, accuracy, is a dichotomous variable. Maternal report of the number of ANC visits either matched with the categorical number of ANC visits observed (the gold standard), indicating accuracy, or it did not match (not accurate). Relative risk of accurately reporting was calculated because accurate reports were not rare outcomes; 38% of women recalled the number of ANC visits accurately according to the categorical definition described previously (1-3 versus 4+ visits; or 1-3 visits, 4 visits, 5-6 visits, and more than 6 visits). Covariates related to maternal characteristics included maternal age, maternal education, number of prior live births, and household SES. All covariates were included in the adjusted model. Maternal age was dichotomized into younger or older than 25 years. Any education was compared to no education and any previous live birth was compared to no previous live birth. The household SES variable was constructed based on family-owned land, animals and household items and housing infrastructures like types of cooking fuels, toilet, and water sources. Housing characteristics were assigned scores and summed up for each woman. The total score was divided by the number of non-missing variables and separated into quartiles. Time between the postpartum interview and the last ANC observation was dichotomized to more or less than 1 year after examining its locally weighted scatterplot smoother (LOWESS) versus report accuracy. The intention was to interview all women at around 6 months postpartum. In practice,

For peer review only - http://bmjopen.bmj.com/site/about/guidelines.xhtml

we did not know when they would deliver, so scheduled their postpartum visit 12 months after their first ANC visit if this was in the 1st or 2nd trimester. If the first ANC visit was in the 3rd trimester, we scheduled the postpartum visit 6 months after the first ANC visit. The time between the last ANC visit and the postpartum visit would be somewhat longer than 6 months since the last ANC visit could occur several months before birth. The observed total number of ANC visits was classified as 1-3, 4-7, and 8 or more using the LOWESS curve. Both LOWESS curves appeared linear in segments with a knot at approximately 1 year and knots at the 4th and 8th visits. A p-value less than 0.05 was considered statistically significant.

All analyses were conducted using Stata version 17.0 (StatCorp).

Ethical Review

The Institutional Review Board of the Johns Hopkins Bloomberg School of Public Health and the Nepal Health Research Council approved the parent study.

Patient and public involvement

Study participants were not involved in the design, recruitment, conduct, or dissemination of this research. The 28-item checklist used for direct observation of the first and all subsequent ANC visits was reviewed by a local community advisory board in Nepal before the start of the study, but the public had no other part in the development or implementation of this study. There are no plans to disseminate results to the participants or community, aside from the local study staff who reside in the community.

RESULTS

Among the 441 women enrolled in the study, seven were lost to follow-up due to migration out of the study area and were not available for the postpartum interview. There was no difference between the background characteristics of the participants who were lost to follow-up and those who stayed in the study. Thirty-two women were excluded from the validation analysis because of their birth

For peer review only - http://bmjopen.bmj.com/site/about/guidelines.xhtml

outcomes (not a live birth). At the time of the study, in the DHS, women with a pregnancy not resulting in a live birth were not asked the question about number of ANC visits (although more recent DHS do). 402 women met the Nepal DHS sampling criteria and were included in the analysis. Among the 402 women, 228 reported receiving ANC at least once from non-study facilities, leaving 174 women with complete ANC observation by the study team. Figure 1 shows the flowchart of participants.

Table 1. Characteristics of participants with live births[1]

Characteristic	Observed all ANC visits (N=174) Mean (SD)	Observed all ANC visits (N=174) Range	Received ANC between observations (N=228) Mean (SD)	Received ANC between observations (N=228) Range	Two sample t-test p-value	Total (N=402) Mean (SD)	Total (N=402) Range
Woman's age, years	22.7 (4.4)	16-41	22.3 (4.1)	16-35	0.318	22.5 (4.2)	16-41
Total number of ANC visits observed	3.4 (2.1)	1-10	5.6 (2.3)	2-14	<0.01	4.7 (2.5)	1-14
Number of months between last ANC observation and postpartum interview	11.2 (3.2)	3-21	9.1 (2.5)	3-17	<0.01	10.0 (3.0)	3-21

	Observed all ANC visits (N=174) n (%)	Received ANC between observations (N=228) n (%)	Chi-square p-value	Total (N=402) n (%)
4 quantiles of SES				
1	76 (43.7)	72 (31.6)	<0.01	148 (36.8)
2	36 (20.7)	35 (15.4)		71 (17.7)
3	43 (24.7)	83 (36.4)		126 (31.3)
4	19 (10.9)	38 (16.7)		57 (14.2)
Is this the woman's first pregnancy?				
No	133 (76.4)	143 (62.7)	<0.01	276 (68.7)
Yes	41 (23.6)	85 (37.3)		126 (31.3)
Did the woman receive any years of education?				
No	121 (69.5)	119 (52.2)	<0.01	240 (59.7)
Yes	53 (30.5)	109 (47.8)		162 (40.3)
Trimester at enrollment				
1-3 months	63 (36.2)	107 (46.9)	0.043	170 (42.3)
4-6 months	106 (60.9)	119 (52.2)		225 (56.0)
7-9 months	5 (2.9)	2 (0.9)		7 (1.7)

For peer review only - http://bmjopen.bmj.com/site/about/guidelines.xhtml

Frequency of ANC visits				
1-3 visits	103 (59.2)	39 (17.1)	<0.01	142 (35.3)
4 visits	25 (14.4)	48 (21.1)		73 (18.2)
5-6 visits	31 (17.8)	71 (31.1)		102 (25.4)
More than 6 visits	15 (8.6)	70 (30.7)		85 (21.1)

[1] ANC, antenatal care; SES, socioeconomic status.

Table 1 summarizes the maternal characteristics of women who attended the postpartum interview and had a live birth outcome. The age of women ranged from 16 to 41 years, with a mean age of 22.5. There was no significant age difference between women who sought ANC elsewhere and those who did not. The observed total number of ANC visits ranged from 1 to 14. On average, women attended 4.7 ANC visits during their pregnancy. The number of ANC visit was higher among women who sought ANC in non-study facilities. About 65% of women attended four or more ANC visits, the majority of which (72.7%) were women who reported receiving ANC from non-study clinics between observations at the study clinics. About 60% of women had not received any education. Women who received ANC from non-study clinics were more educated and had higher SES.

Bland-Altman plots were constructed to compare ANC visit frequencies as observed by the gold standard and reported by maternal recall (Supplemental Figure 1). In the entire cohort, the mean number of ANC visits observed was 4.7 (SD = 2.5), compared with the reported number of 4.4 visits (SD = 1.6). We observed both over-reporting and under-reporting of number of ANC visits, relative to the number observed (Supplemental Figure 1A). Over-reporting was common among women who had fewer ANC visits, while under-reporting was common among higher ANC frequencies. In the subgroup of women whose ANC was fully observed (Supplemental Figure 1B), the observed mean of total visits was 3.4 (SD = 2.1), while the reported mean was 4.0 (SD = 1.7). The distribution of observed total number of visits was positively skewed, with a long tail of women receiving 8+ visits, while the reported visits were more normally distributed (Supplemental Figure 2 and 3). The disparity between the observed and

For peer review only - http://bmjopen.bmj.com/site/about/guidelines.xhtml

reported distributions implied that women who had less or more than four ANC visits tended to report that they had four visits during pregnancy.

A total of 402 women with live births were included in the validity analysis, as per the Nepal DHS protocol. The validation results from the 402 women are shown in Table 2A. The binary indicator of 4+ visits, which is used for global reporting and tracking, had a sensitivity of 89.2% (95% CI: 84.8, 92.7%) and a specificity of 49.3% (95% CI: 40.8, 57.8%). It showed a moderate level of individual validity (AUC: 0.69; 95% CI:0.65, 0.74) and low population-level bias (IF:1.17). The categorized visit groups, on the other hand, demonstrated poorer validity than the binary indicator in terms of sensitivity, AUC, and IF. In general, sensitivity was low and had a declining trend with more ANC visits. The 1-3 visits category had the highest sensitivity score of 49.3% (95% CI: 40.8, 57.8%). Specificity ranged from 63.5% (95% CI: 58.1, 68.7%) in the 4 visits group to 94.0% (95%CI: 90.8, 96.4%) in the more than 6 visits group. Only the 1-3 visits group showed a moderate level of individual validity (AUC: 0.69; 95% CI: 0.65, 0.74), while other groups all had AUC less than 0.6 but barely better than a random guess. Population-level bias was common in all groups except the 5-6 visits group (IF: 1.10). There was high overestimation of ANC visit frequency in the 4 visits group (IF: 2.12) and underestimation in the other two groups. However, specificity of ANC categories was much better than that of the binary indicator. Specificity ranged from 63.5% (95% CI: 58.1, 68.7%) in the 4 visits group to 94.0% (95%CI: 90.8, 96.4%) in the more than 6 visits group.

Table 2. Validation of maternal report of ANC visits[1]

A. Among women with livebirths (N = 402)

Gold standard vs. reported	Sensitivity (95% CI), %	Specificity (95% CI), %	AUC (95% CI)	"True" coverage (95% CI), %	Estimated survey coverage, %	Inflation factor
No. of ANC visits FANC model (4 or more)	89.2 (84.8, 92.7)	49.3 (40.8, 57.8)	0.69 (0.65, 0.74)	64.7 (59.8, 69.4)	75.6	1.17
1 to 3	49.3 (40.8, 57.8)	89.2 (84.8, 92.7)	0.69 (0.65, 0.74)	35.3 (30.6, 40.2)	24.4	0.69
4	47.9 (36.1, 60.0)	63.5 (58.1, 68.7)	0.56 (0.49, 0.62)	18.2 (14.5, 22.3)	38.6	2.12

For peer review only - http://bmjopen.bmj.com/site/about/guidelines.xhtml

For peer review only

5 to 6	30.4 (21.7, 40.3)	73.0 (67.6, 77.9)	0.52 (0.47, 0.57)	25.4 (21.2, 29.9)	27.9	1.10
More than 6	21.2 (13.1, 31.4)	94.0 (90.8, 96.4)	0.58 (0.53, 0.62)	21.1 (17.3, 25.5)	9.2	0.44

B. Among women with livebirths and fully observed (N = 174)

Gold standard vs. reported	Sensitivity (95% CI), %	Specificity (95% CI), %	AUC (95% CI)	"True" coverage (95% CI), %	Estimated survey coverage, %	Inflation factor
No. of ANC visits FANC model (4 or more)	80.3 (69.1, 88.8)	51.5 (41.1, 61.4)	0.66 (0.59, 0.73)	40.8 (33.4, 48.5)	61.5	1.51
1 to 3	51.5 (41.4, 61.4)	80.3 (69.1, 88.8)	0.66 (0.59, 0.73)	59.2 (51.5, 66.6)	38.5	0.65
4	40.0 (21.1, 61.3)	14.4 (9.5, 20.5)	0.54 (0.43, 0.64)	14.4 (9.5, 20.5)	33.3	2.32
5 to 6	19.4 (7.5, 37.5)	80.4 (73.0, 86.6)	0.50 (0.42, 0.58)	17.8 (12.4, 24.3)	19.5	1.10
More than 6	20.0 (4.1, 48.1)	92.5 (87.2, 96.0)	0.56 (0.46, 0.67)	8.6 (4.9, 13.8)	8.6	1.00

[1] ANC, antenatal care; FANC, focused antenatal care.

When considering only the subgroup with complete observation (Table 2B), the binary indicator 4+ visits still had better sensitivity and AUC than the multi-categorical variable. However, the IF increased to 1.51, indicating overestimation of four or more ANC visits at the population level. Sensitivity remained low among all visit categories and had the same decreasing trend in the overall population. Specificity was relatively similar. Individual validity was still highest but not very good in the 1-3 visits category (AUC: 0.66; 95% CI: 0.59, 0.73) while others were no better than a random guess. However, two groups demonstrated great population-level validity. The 5-6 and more than 6 visits groups now had IFs of 1.10 and 1.00 respectively, indicating low population-level bias. Overestimation still existed in the 4 visits group with an IF of 2.32.

Figure 2 is an IF graph created based on the sensitivity, specificity, and true population coverage of the binary indicator (4+ visits) among women with live births. The difference between the observed and reported coverage is illustrated by the vertical red line. As outlined in the graph, maternal report tends to overestimate the number of ANC visits at lower numbers of visits, but underestimates at higher numbers of visits. Even in subgroups with IF close to 1.00, the survey estimation could greatly deviate from the true measurement depending on the true coverage of ANC4+.

For peer review only

For peer review only - http://bmjopen.bmj.com/site/about/guidelines.xhtml

Table 3. Maternal characteristics associated with report accuracy[1]

	n (%)	Unadjusted RR (95% CI)	Adjusted RR (95% CI)
Any education	162 (40.3)	0.97 (0.75, 1.25)	0.96 (0.73, 1.24)
Any previous live birth	126 (31.3)	1.18 (0.92, 1.53)	1.22 (0.93, 1.61)
Age >=25	122 (30.4)	0.89 (0.67, 1.18)	0.92 (0.68, 1.26)
SES quartiles (ref: first)			
2	71 (17.6)	0.93 (0.65, 1.35)	0.93 (0.64, 1.34)
3	126 (31.3)	1.05 (0.79, 1.41)	0.98 (0.72, 1.31)
4	57 (14.2)	0.81 (0.52, 1.24)	0.77 (0.50, 1.21)
>1 yr since last ANC observation	76 (18.9%)	1.17 (0.87, 1.57)	0.91 (0.67, 1.24)
Number of ANC visit (ref: 1 to 3)			
4 to 7	200 (49.8)	0.70 (0.54, 0.90)[2]	0.66 (0.51, 0.87)[2]
8 or more	60 (14.9)	0.51 (0.32, 0.81)[2]	0.48 (0.30, 0.77)[2]

[1] ANC, antenatal care; SES, socioeconomic status.
[2] $P < 0.05$

Among the 402 women with live births, only 85 (21.1%) women's report matched exactly the ANC number as observed by the gold standard. Using categorical accuracy, 154 (38.3%) women reported correctly. The categorical accuracy rate was slightly higher in those who did not seek ANC elsewhere (41.4%) compared to those who visited other facilities (36.0%), but the difference was not statistically significant. Maternal characteristics such as education, previous birth, age, and SES were not associated with reporting accuracy (Table 3). The number of total ANC visits had the strongest association with maternal report accuracy, with increasing number of ANC visits associated with lower reporting accuracy. The unadjusted risk for women who received 4-7 and 8 or more ANC visits, compared to the 1-3 visit group, was 0.70 (95% CI: 0.54, 0.90) and 0.51 (95% CI: 0.32, 0.81) respectively. After adjusting for other variables, both RR decreased slightly to 0.66 (95% CI: 0.51, 0.87) and 0.48 (95% CI: 0.30, 0.77) and remained significant. This suggested that women with 4-7 ANC visits were 34% less likely to report this information correctly during household surveys, and women who had 8 or more ANC visits were 52% less likely to recall correctly, comparing to those attended 1-3 ANC visits.

DISCUSSION

For peer review only - http://bmjopen.bmj.com/site/about/guidelines.xhtml

This study examined the validity of maternal report of total number of ANC visits during pregnancy in rural Nepal using data from direct ANC observation. To our knowledge, it is the first study that validates number of ANC visits as an indicator in ANC coverage measurement. In general, individual-level validity was poor among women with four or more ANC visits and moderate among women with fewer ANC visits when using categorized ANC visits as an indicator, but was higher for the binary ANC4+ indicator. The validation results of the multi-categorial variable showed that four ANC visits was often overreported. Population-level bias seemed to be low among women with a higher number of ANC visits, but the survey question greatly overestimates the true coverage at lower prevalence and underestimates it at higher prevalence. Less than half of women recalled the exact number of visits correctly during the postpartum visit. Reporting accuracy was found to be negatively associated with the total number of ANC visits during pregnancy but was not associated with maternal characteristics such as age, education, parity, and SES. The recall period was also not associated with accuracy of recall but there was not a wide range of recall times to examine this variable.

These validation results suggest some bias in household surveys that report number of ANC visits and that report ANC4+. At the population level, 1-3 visits were underreported, but having had four ANC visits was highly overreported among the multi-categorical variable. This might be due to Nepal's guideline on ANC, which was based on the FANC model, that may have introduced bias in household surveys with women more likely to report the norm or expected number of visits for which they would be paid through the cash incentive system. Only the 1-3 visits groups showed a moderate level of individual validity, which was consistent with the regression results where more ANC visits was associated with less accurate self-report. Besides the participant's ability to recall correctly, cognitive and situational issues are usually the two factors associated with self-report validity [18]. In this case, the language used during the postpartum interview, which was specifically designed to resemble that used in the DHS, could be misunderstood. A study of cognitive testing of questions about antenatal care

For peer review only - http://bmjopen.bmj.com/site/about/guidelines.xhtml

suggested that overreporting and underreporting may be related to the definition of an ANC visit [19]. ANC visits are meant to be regular preventive checkups in pregnancy. However, if a woman came for care because she was sick, this would be counted as an ANC visit in the gold standard observed count but might not be counted as an ANC checkup visit by the woman at the time of recall six months postpartum [19]. Social desirability bias is the inclination of people to report more socially desired activities than they actually performed (overreporting) or understate undesirable attributes (underreport) [20]. In the study scenario, women who had less than four ANC tended to report more and meet the social standard in front of the interviewer and sometimes the presence of their husbands, resulting in the underreporting of 1-3 visits group and overreporting of the 4 visits group. The low population-level bias here may be explained by the low prevalence of 5-6 visits due to a low number of false negatives. People who had more than six ANC visits seemed to underreport their receipt of care. This might be attributed to the respondents' inability to recall higher number of visits. ANC visits are often concentrated towards the end of pregnancy. Women might have conflated the visits in their minds and recalled a lower number. A study of social desirability bias was undertaken as part of this validity study. It showed very little social desirability bias but did show situational bias associated with whether family members or others were present during the postpartum interview (Thorne-Lyman, unpublished data, 2023). It was found that the presence of any adult at the interview is associated with greater risk of overestimation of ANC frequency, with the presence of the husband being the most influential (Thorne-Lyman, unpublished data, 2023).

There have been several yet limited studies on the validity of health indicators in coverage measurement. This paper contributes to the current body of validation studies and factors related to the accuracy of ANC self-report. One similar study evaluated the coverage rate of intermittent preventive treatment during pregnancy based on mother's recall in Benin, Ghana, Malawi, and Tanzania [21]. It was found that compared to ANC card data (the gold standard), recalled data in household surveys were

For peer review only

valid [21]. Sensitivity and specificity of self-report were generally higher than that in our study, and notably, the AUC of reported measurements from all four countries was higher than 0.8 [21]. One potential reason for the different conclusions between the two studies could be that the recommended frequency for intermittent preventive treatment (at least 3 times) is lower than that of ANC, resulting in less variation in the total number of ANC visits and making it easier to recall correctly during household surveys. Additionally, ANC cards were used as the gold standard in their case instead of health facility records [21]. This could bias the results as women's self-reported validity might be associated with their ability to keep health records, which makes ANC cards not an optimal source for verification. Those who had a card would be more likely to read the card and be reminded of the number of visits they had.

In this study, the binary indicator 4+ visits performed better than multi-categorical indicators (1-3 visits, 4 visits, 5-6 visits, and more than 6 visits) in terms of both individual- and population-level validity. In previous studies, dichotomous indicators often possessed higher validity than counts or frequency for the same intervention in household surveys. For example, the NNIPS study on iron-folic acid found that report of "any iron-folic acid receipt" demonstrated better individual validity and very low population-level bias compared to specific tablet counts [14]. However, in this study, the prevalence of receipt of any iron-folic acid was very high (over 95%), which is likely the primary reason for higher validity and low bias. Furthermore, in a study comparing national household survey and health facility service statistics in Uganda, there was considerable agreement between the two data sources for skilled attendance at birth and at least four ANC visits [22]. However, if the number of ANC visits were dichotomized at eight times, the validity might not be better than that of categorical indicators. Many studies also have found that report accuracy was associated with the length of recall period, where accuracy decreases with extended duration of recall [23-25], but such relation was not seen in the NNIPS studies.

For peer review only - http://bmjopen.bmj.com/site/about/guidelines.xhtml

A strength of this study is that the gold standard used in validation was through direct observation by trained field workers. Study observers were all trained to reach a standard level of validity before working at the study sites, which makes it a more objective and reliable source for verification than secondary databases. A second strength is that the study had a reasonable length of recall period, not as long as DHS but longer than many other studies. Other validation studies use recall periods of less than six months or even exit interviews to validate maternal report. One of the main limitations is that the study only considered women who presented for their first ANC at public health posts. Women who never attend ANC or those who do not go to public facilities were not captured through the study, but they may have characteristics that influence the overall self-report validity. Another limitation is that the study was unable to observe women if they went to other facilities for ANC. Subgroup analysis with just those women with all their ANC visits observed was conducted for more rigorous validation results. However, these measures were dependent on the women's ability to recall and report their care-seeking behavior at other clinics. Lastly, the study was limited to only five health posts across two municipalities. Thus, the study result may not be generalizable to all women in the 20 Sarlahi municipalities, or Nepal's rural population in general.

CONCLUSION

The DHS surveys are used in many countries to track progress in provision of ANC services and quality of maternal and newborn care. While the number of ANC visits does not imply quality of care, it is an important first step. If women are unable to accurately recall the number of ANC visits attended, this measure of progress is not very useful and ways to measure number of visits should be reconsidered. In general, the number of ANC visits as asked during DHS or household surveys, was not accurately recalled, although ANC4+ (a major marker of ANC coverage progress) recalled better than if ANC was more finely categorized. For women with more ANC visits than the standard of four (for Nepal at the time of this study), women tended to underreport the number of ANC visits. With the change

For peer review only - http://bmjopen.bmj.com/site/about/guidelines.xhtml

from four or more to eight or more ANC visits as the standard, approaches to improving recall should be identified and implemented.

For peer review only

For peer review only - http://bmjopen.bmj.com/site/about/guidelines.xhtml

Author Contributions: MM and JK designed the study. TPL, SKK, SLC, JK and EB contributed to the implementation of the study. XX conducted the analyses and wrote the first draft of the manuscript. JK submitted edits to the manuscript. MM, JK, TPL, SKK, EB and SCL all have read and approved the final version of the manuscript. JK acted as the guarantor.

Funding: This research was funded by the Bill and Melinda Gates Foundation (grant number OPP1172551).

Competing interests: None declared.

Patient consent for publication: Not applicable.

Ethics approval: The Institutional Review Board of the Johns Hopkins Bloomberg School of Public Health and the Nepal Health Research Council approved the parent study.

Data availability statement: Data are available upon reasonable request.

For peer review only

REFERENCES

1. World Health Organization. Trends in maternal mortality 2000 to 2017: Estimates by WHO, UNICEF, UNFPA, World Bank Group and the United Nations Population Division. Geneva: WHO, 2019.
2. UNICEF DATA. Neonatal mortality [online]. January 2023. https://data.unicef.org/topic/child-survival/neonatal-mortality/ (accessed 20 April 2023).
3. World Health Organization. Maternal mortality [online]. February 2023. https://www.who.int/news-room/fact-sheets/detail/maternal-mortality (accessed 20 April 2023).
4. Wondemagegn AT, Alebel A, Tesema C, Abie W. The effect of antenatal care follow-up on Neonatal Health Outcomes: A systematic review and meta-analysis. Public Health Reviews. 2018;39(1). doi:10.1186/s40985-018-0110-y
5. Tekelab T, Chojenta C, Smith R, Loxton D. The impact of antenatal care on neonatal mortality in sub-Saharan africa: A systematic review and meta-analysis. PLOS ONE. 2019;14(9). doi:10.1371/journal.pone.0222566
6. Aryal KK, Sharma SK, Khanal MN, et al. Maternal health care in Nepal: Trends and determinants (English). DHS Further Analysis Reports No. 118. The DHS Program, 2019.
7. Khatri RB, Durham J, Assefa Y. Utilisation of quality antenatal, delivery and postnatal care services in Nepal: An Analysis of Service Provision Assessment. Globalization and Health. 2021;17(1). doi:10.1186/s12992-021-00752-x
8. Powell-Jackson T, Neupane BD, Tiwari S, et al. The impact of Nepal's National Incentive Programme to promote safe delivery in the district of Makwanpur. Innovations in Health System Finance in Developing and Transitional Economies. 2009;221–49. doi:10.1108/s0731-2199(2009)0000021012
9. Ensor T, Bhatt H, Tiwari S. Incentivizing Universal Safe Delivery in Nepal: 10 years of experience. Health Policy and Planning. 2017;32(8):1185–92. doi:10.1093/heapol/czx070
10. Ministry of Health and Population. National Medical Standard for maternal and newborn care volume III. Nepal: Ministry of Health and Population, 2020.
11. Ministry of Health. Nepal Demographic and Health Survey 2016. Kathmandu, Nepal: Ministry of Health, Nepal, 2017.
12. Bryce E, Katz J, Pema Lama T, et al. Antenatal care processes in rural southern Nepal: Gaps in and quality of service provision—a cohort study. BMJ Open. 2021;11(12). doi:10.1136/bmjopen-2021-056392
13. Bryce E, Katz J, Heidkamp R, et al. Validation of maternal report of nutrition-related interventions and counselling during antenatal care in southern Nepal. Maternal & Child Nutrition. 2021;18(2). doi:10.1111/mcn.13303
14. Bryce E, Munos M, Lama TP, et al. Validation of maternal report of receipt of iron–folic acid supplementation during antenatal care in rural southern Nepal. The Journal of Nutrition. 2022;152(1):310–8. doi:10.1093/jn/nxab336
15. Central Bureau of Statistics, Government of Nepal. National Population and Housing Census 2011. Kathmandu, Nepal: Central Bureau of Statistics, Government of Nepal, 2012.
16. Wee DW, Mullany LC, Katz J, et al. Pregnancy-related mortality in southern Nepal between 2001 and 2006: Independent estimates from a prospective, population-based cohort and a direct Sisterhood survey. American Journal of Epidemiology. 2010;172(7):855–60. doi:10.1093/aje/kwq207
17. Munos MK, Blanc AK, Carter ED, et al. Validation studies for population-based intervention coverage indicators: Design, analysis, and interpretation. Journal of Global Health. 2018;8(2). doi:10.7189/jogh.08.020804
18. Brener ND, Billy JOG, Grady WR. Assessment of factors affecting the validity of self-reported health-risk behavior among adolescents: Evidence from the scientific literature. Journal of Adolescent Health. 2003;33(6):436–57. doi:10.1016/s1054-139x(03)00052-1

For peer review only

19. L-Thorne-Lyman A, Lama TP, Heidkamp RA, et al. Cognitive testing of questions about antenatal care and nutrition interventions in southern Nepal. Social Science & Medicine. 2022;311:115318. doi:10.1016/j.socscimed.2022.115318
20. Latkin CA, Edwards C, Davey-Rothwell MA, et al. The relationship between social desirability bias and self-reports of health, substance use, and social network factors among urban substance users in Baltimore, Maryland. Addictive Behaviors. 2017;73:133–6. doi:10.1016/j.addbeh.2017.05.005
21. Hansen N, Youll S, Florey L, Taylor C. Intermittent preventive treatment of malaria in pregnancy coverage estimates from population-based surveys: Reliability of Women's recall among women with ANC cards. The American Journal of Tropical Medicine and Hygiene. 2021;105(2):472–6. doi:10.4269/ajtmh.20-1296
22. Simmons EM, Singh K, Mpiima J, et al. Assessing coverage of essential maternal and child health interventions using health-facility data in Uganda. Population Health Metrics. 2020;18(1). doi:10.1186/s12963-020-00236-x
23. Zimmerman LA, Shiferaw S, Seme A, et al. Evaluating consistency of recall of maternal and newborn care complications and intervention coverage using PMA panel data in SNNPR, Ethiopia. PLOS ONE. 2019;14(5). doi:10.1371/journal.pone.0216612
24. D'Souza-Vazirani D, Minkovitz CS, Strobino DM. Validity of maternal report of acute health care use for children younger than 3 years. Archives of Pediatrics & Adolescent Medicine. 2005;159(2). doi:10.1001/archpedi.159.2.167
25. Kanyangarara M, Katz J, Munos MK, et al. Validity of self-reported receipt of iron supplements during pregnancy: Implications for coverage measurement. BMC Pregnancy and Childbirth. 2019;19(1). doi:10.1186/s12884-019-2247-1

For peer review only

For peer review only - http://bmjopen.bmj.com/site/about/guidelines.xhtml

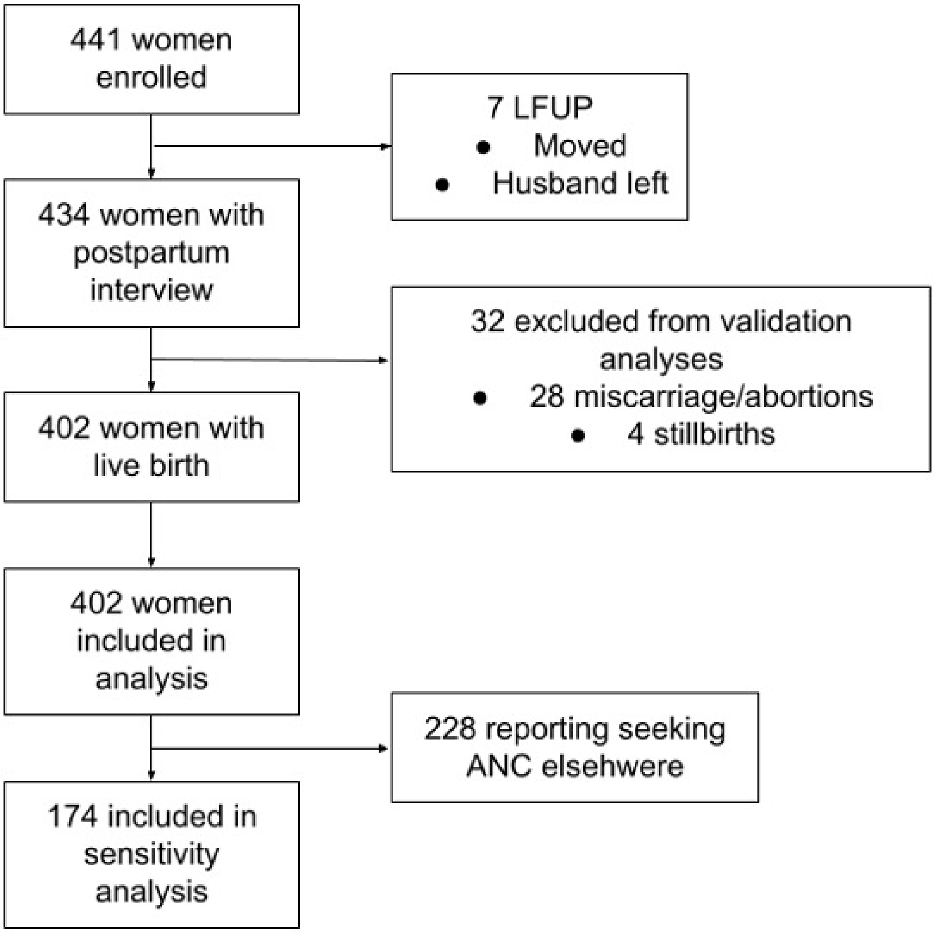

Figure 1. Participant flowchart. ANC, antenatal care; LFUP, lost to follow-up.

102x102mm (144 x 144 DPI)

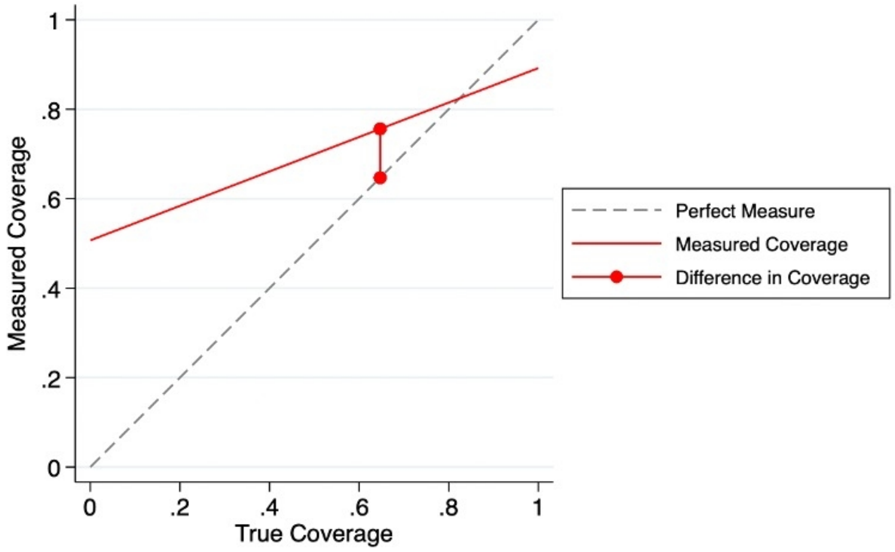

Figure 2. Ture coverage compared with measured coverage for four or more ANC visits.

147x98mm (144 x 144 DPI)

SUPPLEMENTAL TABLES AND FIGURES

Supplemental Table 1. Characteristics of participants with postpartum interview

	Observed all ANC visits (N=204)		Received ANC between observations (N=230)		Two sample t-test p-value	Total (N=434)	
Characteristic	Mean (SD)	Range	Mean (SD)	Range		Mean (SD)	Range
Woman's age, years	22.8 (4.3)	16 to 41	22.3 (4.0)	16 to 35	0.184	22.5 (4.2)	16 to 41
Total number of ANC visits observed	3.2 (2.0)	1 to 10	5.6 (2.3)	2 to 14	<0.01	4.5 (2.5)	1 to 14
Number of months between last ANC observation and postpartum interview	11.6 (3.3)	3 to 22	9.1 (2.5)	3 to 17	<0.01	10.3 (3.2)	3 to 22

	Observed all ANC visits (N=204)		Received ANC between observations (N=230)		Chi-square p-value	Total (N=434)	
Most recent pregnancy outcome							
Miscarriage/abortion	27	13.2%	1	0.4%	<0.01	28	6.5%
Stillbirth	3	1.5%	1	0.4%		4	0.9%
At least one live birth	174	85.3%	228	99.1%		402	92.6%
4 quantiles of SES							
1	94	46.1%	73	31.7%	<0.01	167	38.5%
2	39	19.1%	35	15.2%		74	17.1%
3	48	23.5%	84	36.5%		132	30.4%
4	23	11.3%	38	16.5%		61	14.1%
Is this the woman's first pregnancy?							
No	153	75.0%	145	63.0%	<0.01	298	68.7%
Yes	51	25.0%	85	37.0%		136	31.3%
Did the woman receive any years of education?							
No	139	68.1%	120	52.2%	<0.01	259	59.7%
Yes	65	31.9%	110	47.8%		175	40.3%
Trimester at enrollment							
1-3 months	81	39.7%	109	47.4%	0.1	190	43.8%
4-6 months	117	57.4%	119	51.7%		236	54.4%
7-9 months	6	2.9%	2	0.9%		8	1.8%

For peer review only

Supplemental Figure 1. Agreement between numbers of ANC visits observed at health posts and maternal report at postpartum interview in (A) the entire cohort (n = 402) and (B) the subgroup with all ANC observed (n = 174).

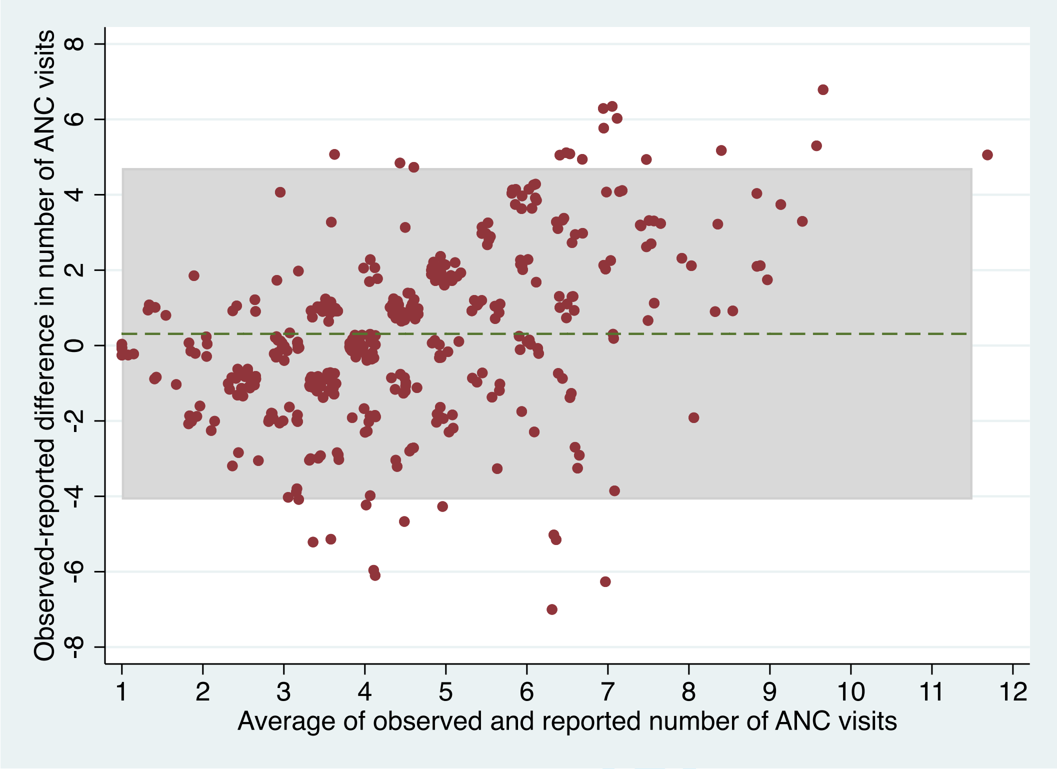

(A)

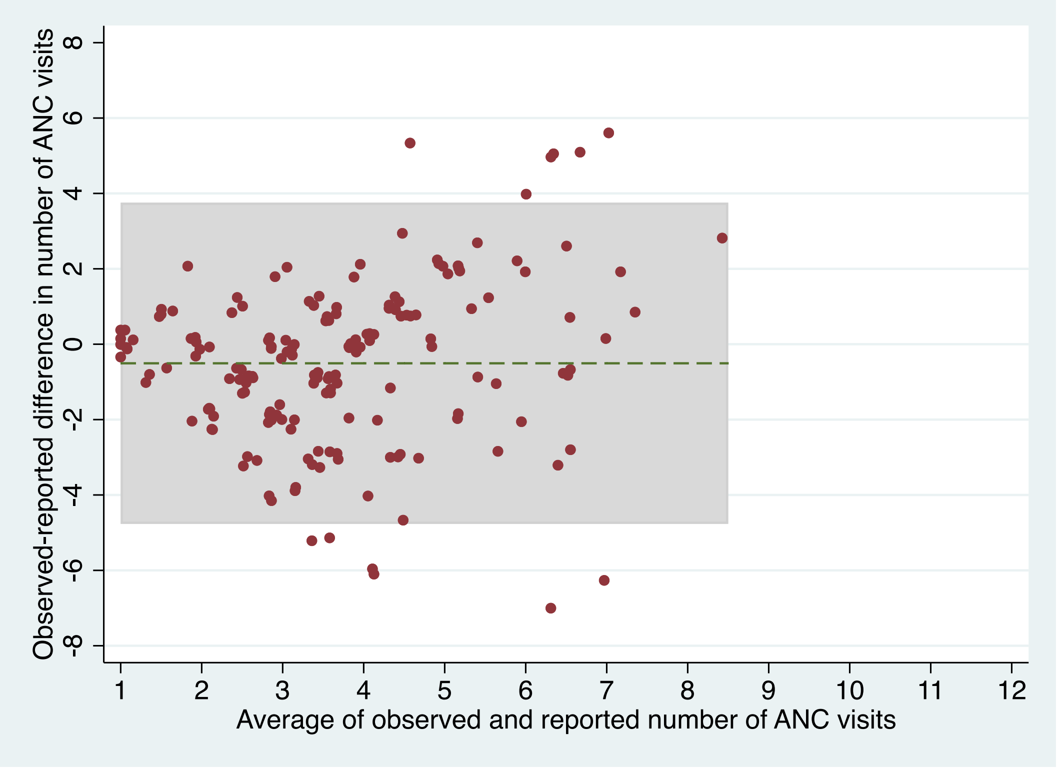

(B)

Supplemental Figure 2. Histogram of observed number of ANC visits (n = 402).

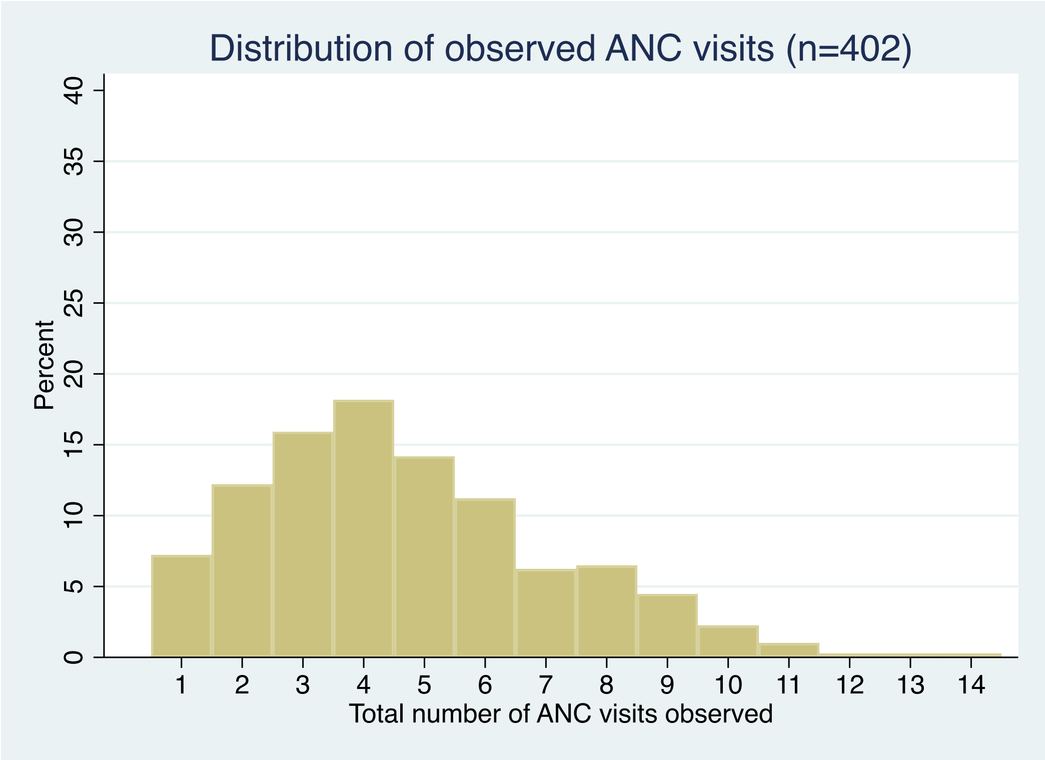

Supplemental Figure 3. Histogram of reported number of ANC visits (n = 402).

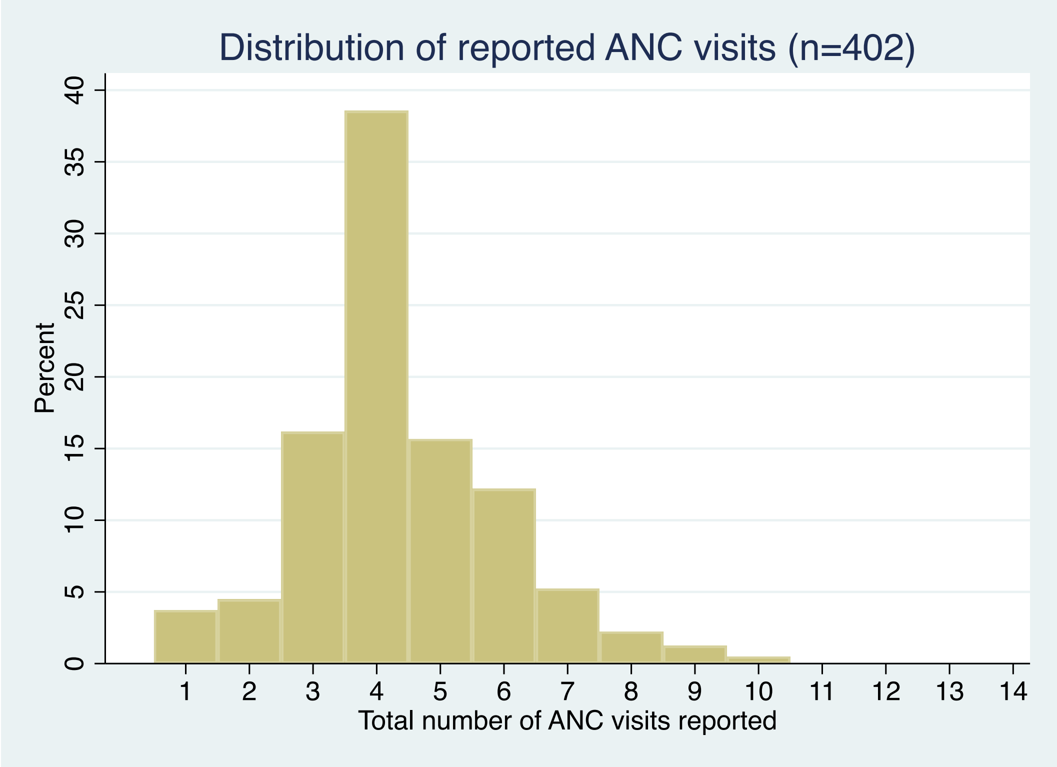

STROBE Statement—Checklist of items that should be included in reports of ***cohort studies***

	Item No	Recommendation	Page No
**Title and abstract**	1	(*a*) Indicate the study's design with a commonly used term in the title or the abstract<br>(*b*) Provide in the abstract an informative and balanced summary of what was done and what was found	1-2
**Introduction**			
Background/rationale	2	Explain the scientific background and rationale for the investigation being reported	4-5
Objectives	3	State specific objectives, including any prespecified hypotheses	5
**Methods**			
Study design	4	Present key elements of study design early in the paper	7
Setting	5	Describe the setting, locations, and relevant dates, including periods of recruitment, exposure, follow-up, and data collection	5-6
Participants	6	(*a*) Give the eligibility criteria, and the sources and methods of selection of participants. Describe methods of follow-up<br>(*b*) For matched studies, give matching criteria and number of exposed and unexposed	6-7
Variables	7	Clearly define all outcomes, exposures, predictors, potential confounders, and effect modifiers. Give diagnostic criteria, if applicable	9
Data sources/ measurement	8*	For each variable of interest, give sources of data and details of methods of assessment (measurement). Describe comparability of assessment methods if there is more than one group	7
Bias	9	Describe any efforts to address potential sources of bias	7
Study size	10	Explain how the study size was arrived at	8
Quantitative variables	11	Explain how quantitative variables were handled in the analyses. If applicable, describe which groupings were chosen and why	8-9
Statistical methods	12	(*a*) Describe all statistical methods, including those used to control for confounding<br>(*b*) Describe any methods used to examine subgroups and interactions<br>(*c*) Explain how missing data were addressed<br>(*d*) If applicable, explain how loss to follow-up was addressed<br>(*e*) Describe any sensitivity analyses	9-10
**Results**			
Participants	13*	(a) Report numbers of individuals at each stage of study—eg numbers potentially eligible, examined for eligibility, confirmed eligible, included in the study, completing follow-up, and analysed<br>(b) Give reasons for non-participation at each stage<br>(c) Consider use of a flow diagram	10-11
Descriptive data	14*	(a) Give characteristics of study participants (eg demographic, clinical, social) and information on exposures and potential confounders<br>(b) Indicate number of participants with missing data for each variable of interest<br>(c) Summarise follow-up time (eg, average and total amount)	11-12
Outcome data	15*	Report numbers of outcome events or summary measures over time	12-14

For peer review only - http://bmjopen.bmj.com/site/about/guidelines.xhtml

Main results	16	(*a*) Give unadjusted estimates and, if applicable, confounder-adjusted estimates and their precision (eg, 95% confidence interval). Make clear which confounders were adjusted for and why they were included<br>(*b*) Report category boundaries when continuous variables were categorized<br>(*c*) If relevant, consider translating estimates of relative risk into absolute risk for a meaningful time period	15
Other analyses	17	Report other analyses done—eg analyses of subgroups and interactions, and sensitivity analyses	14
**Discussion**			
Key results	18	Summarise key results with reference to study objectives	16
Limitations	19	Discuss limitations of the study, taking into account sources of potential bias or imprecision. Discuss both direction and magnitude of any potential bias	19
Interpretation	20	Give a cautious overall interpretation of results considering objectives, limitations, multiplicity of analyses, results from similar studies, and other relevant evidence	16-18
Generalisability	21	Discuss the generalisability (external validity) of the study results	19
**Other information**			
Funding	22	Give the source of funding and the role of the funders for the present study and, if applicable, for the original study on which the present article is based	21

*Give information separately for exposed and unexposed groups.

Note: An Explanation and Elaboration article discusses each checklist item and gives methodological background and published examples of transparent reporting. The STROBE checklist is best used in conjunction with this article (freely available on the Web sites of PLoS Medicine at http://www.plosmedicine.org/, Annals of Internal Medicine at http://www.annals.org/, and Epidemiology at http://www.epidem.com/). Information on the STROBE Initiative is available at http://www.strobe-statement.org.

For peer review only
